# Impaired AKT signaling and lung tumorigenesis by PIERCE1 ablation in KRAS-mutant non-small cell lung cancer

**DOI:** 10.1038/s41388-020-01399-5

**Published:** 2020-07-29

**Authors:** Jae-il Roh, Jaehoon Lee, Young-Hoon Sung, Jahyun Oh, Do Young Hyeon, Yujin Kim, Seungeon Lee, Sushil Devkota, Hye Jeong Kim, Bomin Park, Taewook Nam, Yaechan Song, Yonghwan Kim, Daehee Hwang, Han-Woong Lee

**Affiliations:** 1 Department of Biochemistry, College of Life Science and Biotechnology, Yonsei University, Seoul, Korea; 2 Department of Convergence Medicine, University of Ulsan College of Medicine, Seoul, Korea; 3 Department of Biological Sciences, Seoul National University, Seoul, Korea; 4 Department of Cell and Developmental Biology, University of California, San Diego, CA 92093 USA; 5 Department of Life Systems, Sookmyung Women’s University, Seoul, Korea

**Keywords:** Non-small-cell lung cancer, Cell signalling

## Abstract

KRAS-mutant non-small cell lung cancer (NSCLC) is a major lung cancer subtype that leads to many cancer-related deaths worldwide. Although numerous studies on KRAS-mutant type NSCLC have been conducted, new oncogenic or tumor suppressive genes need to be detected because a large proportion of NSCLC patients does not respond to currently used therapeutics. Here, we show the tumor-promoting function of a cell cycle-related protein, PIERCE1, in KRAS-mutant NSCLC. Mechanistically, PIERCE1 depletion inhibits cell growth and AKT phosphorylation (pAKT) at S473, which is particularly observed in KRAS-mutant lung cancers. Analyses of AKT-related genes using microarray, immunoblotting, and real-time quantitative PCR indicated that PIERCE1 negatively regulates the gene expression of the AKT suppressor, TRIB3, through the CHOP pathway, which is a key regulatory pathway for TRIB3 expression. Similarly, in vivo analyses of PIERCE1 depletion in the KRAS mutation-related lung cancer mouse models revealed the suppressive effect of PIERCE1 knockout in urethane- and KRAS^G12D^-induced lung tumorigenesis with decreased pAKT levels observed in the tumors. Tissue microarrays of human lung cancers indicated the expression of PIERCE1 in 83% of lung cancers and its correlation with pAKT expression. Thus, we illustrate how PIERCE1 depletion may serve as a therapeutic strategy against KRAS-mutant NSCLC and propose the clinical benefit of PIERCE1.

## Introduction

PIERCE1, p53-induced expression in retinoblastoma (RB)-null cells 1, also known as C9orf116 and RbEST47, was discovered as a RB-regulated novel p53 target gene associated with cell cycle in mice, and it is predominantly expressed in the brain, kidneys, and lungs of humans [1, 2]. PIERCE1 deficiency causes developmental abnormalities and embryonic lethality in approximately half of all PIERCE1 homozygous knockout (KO) mice [3]. Among the surviving PIERCE1 KO mice, approximately 40% have *situs inversus totalis*, a condition in which all organs are reversed from their normal positions [3]. PIERCE1 is induced by the elimination of RB, a phenomenon which is completely blocked by p53 KO in mouse embryonic fibroblasts [1, 2]. Genotoxic stresses, such as ultraviolet C (UVC), can induce PIERCE1 expression and stabilize its protein by proteasome inhibitor treatment [2]. A recent report showed enhanced cell proliferation with upregulation of cyclin A2 (CCNA2), cyclin D1 (CCND1), and MYC expressions by overexpression of PIERCE1 in rat liver cells [4]. This suggests that PIERCE1 modulates cell cycle transition and proliferation during tumorigenesis.

Lung cancer is the deadliest form of cancer worldwide [5]. Non-small cell lung cancer (NSCLC) accounts for more than 80% of all lung cancer cases and can be divided into the following three major subtypes: adenocarcinoma, squamous-cell carcinoma, and large-cell carcinoma [6]. Mutations in *KRAS* (25%), *EGFR* (23%), and *EML4-ALK* (5%) genes are present in approximately 50% of all adenocarcinoma patients [7], while other genetic variations have been observed in squamous- and large-cell carcinomas [8]. Mutations in *KRAS* occur mainly at codons 12 or 13, and rarely at other codons, such as codon 61 [9]. Once KRAS is mutated, it constitutively activates its downstream targets, such as MAPK and AKT pathways, resulting in increased cell proliferation and survival [10]. Although a number of inhibitors are currently used against certain types of NSCLCs, KRAS-mutant NSCLC treatment strategies are still markedly limited.

Numerous kinases and phosphatases regulate the phosphorylation of AKT at threonine 308 (T308) and serine 473 (S473) residues. For instance, RAS-mediated activation of PI3K, which phosphorylates phosphatidylinositol-4,5-bisphosphate (PIP2) to phosphatidylinositol-(3,4,5)-trisphosphate (PIP3), induces AKT phosphorylation at T308 through 3-phosphoinositide-dependent kinase-1 (PDK1) [11]. In comparison to T308 phosphorylation, other kinases such as mammalian target of rapamycin complex 2 (mTORC2), tribbles homolog 3 (TRIB3), phosphoinositide-dependent protein kinase-2 (PDK2), and TANK binding kinase-1 (TBK1) are involved in AKT phosphorylation at residue S473 [12, 13]. Phosphorylation at S473 is required for the maximal activation of AKT and stabilization of T308 phosphorylation [11]. AKT is multifunctional and controls cellular survival, growth, proliferation, metabolism, and migration [14] mainly through mTOR complex I (mTORC1), resulting in increased tumorigenesis and drug resistance in various tumor types [15, 16]. Upon activation of mTORC1, its downstream targets such as ribosomal protein S6 kinase (S6K), unc-51-like kinase-1 (ULK1), and eukaryotic translation initiation factor 4E (eIF4E)-binding protein 1 (4EBP1), are phosphorylated resulting in physiological changes [17].

TRIB3, a pseudokinase which belongs to the tribbles family, is important for signaling regulation. TRIB3 interacts with several kinases and transcription factors and is known to suppress cellular division and tumorigenesis [18, 19]. Enhanced tumorigenesis has been observed following genetic inhibition of TRIB3, along with a rise in AKT phosphorylation (pAKT), specifically in the presence of activating mutations in *HRAS* and deletion of *PTEN* [18]. Under specific conditions such as arsenite treatment and endoplasmic-reticulum (ER) stress, transcriptional activation of TRIB3 can be performed through transcription factors, such as activating transcription factor 4 (ATF4)-C/EBP homologous protein (CHOP) [20, 21]. Further investigations have revealed that activation of TRIB3 expression by peroxisome proliferator-activated receptors results in the suppression of AKT pathway in lung and pancreatic cancer cells [22].

Upon accumulation of unfolded proteins in the ER, GRP78 binds to these proteins, leading to the activation of ER membrane proteins including inositol-requiring enzyme 1, protein kinase RNA-like endoplasmic-reticulum kinase (PERK), and ATF6 [23], which in turn activate multiple signaling cascades including autophagy and regulation of protein synthesis to control homeostasis [24]. ATF4 and CHOP, transcription factors activated by ER stress, promote the expression of genes involved in cell death [24]. Moreover, the antitumor effect of ER stress against KRAS-mutant lung cancer suggests a potential novel strategy as a therapeutic target for lung cancer [25]. This study provides insights into a novel and promising therapeutic strategy that inhibits PIERCE1 in KRAS-mutant type NSCLC.

## Results

### PIERCE1 promotes cell growth especially in KRAS-mutant lung cancer

**Fig. 1:**
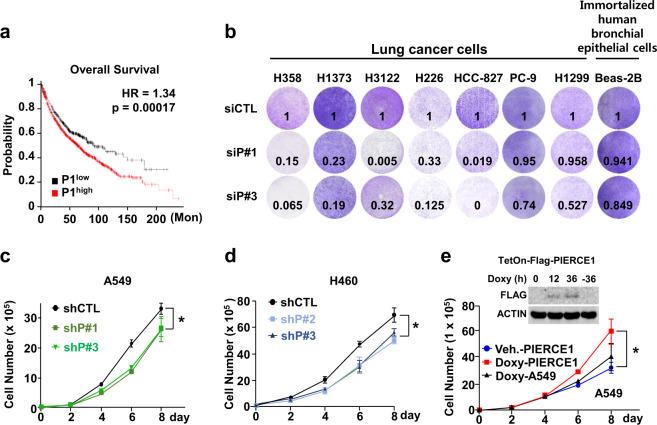
Depletion of PIERCE1 inhibits lung cancer cell growth. **a** Kaplan–Meier plotter analyses of overall survival (OS) in 1926 lung cancer patients with respect to PIERCE1 expression low (black) and high (red) groups. Auto select best cutoff was chosen for the analyses. The hazard ratio (HR) and *P* values were indicated. **b** Monitoring of growth of lung cancer cell lines by crystal violet staining 72 hours after siRNA transfection against control (siCTL) and PIERCE1 (siP#1 and siP#3). Quantitated relative intensities of crystal violet staining of PIERCE1 knockdown (KD) cells to the controls were indicated in the bottom middle of each well. The growth rates of A549 (**c**) and H460 (**d**) cell lines that have stable PIERCE1 KD with shRNA were compared with those of the control on the indicated days. **e** Growth rates of parental (black) and Doxy-inducible PIERCE1 overexpressing A549 cell lines with or without doxycycline treatment (red and blue, respectively) at the indicated days. Error bars are ±SD *P < 0.05.

PIERCE1 is a tumor-associated protein that promotes cell proliferation and controls cell death under stress conditions [2, 4]. The cancer promoting activity and lung-specific expression of PIERCE1 prompted us to examine the link between PIERCE1 and lung cancer. Moreover, ablation of RB, one of the major suppressors of PIERCE1 expression, accelerates tumor progression in lung adenocarcinoma [26], potentiating our hypothesis. We first analyzed overall and progression-free survival rates of total lung cancer cases from the Kaplan–Meier plotter [27]. Patients were divided into two groups according to their PIERCE1 expression levels, i.e., either high (*n* = 1441) or low (*n* = 485). The results showed that patients with low PIERCE1 expression had significantly improved overall and progression-free survival rates compared to patients with high expression levels (Fig. 1a and Supplementary Fig. 1a), suggesting that PIERCE1 might be involved in lung tumorigenesis. To elucidate the influence of PIERCE1 in lung tumorigenesis, cell growth was measured following PIERCE1 knockdown (KD) in lung cancer cell lines. Transient KD of PIERCE1 expression hindered proliferation of five of seven lung cancer cell lines (H358, H1373, H3122, H226, and HCC827); however, no changes were detected in PC-9 and H1299 cell lines. One immortalized human bronchial epithelial cell line, BEAS-2B, also showed no effect upon PIERCE1 KD (Fig. 1b), suggesting that PIERCE1 depletion suppresses cell growth in specific lung cancer cell lines. To confirm this, we generated PIERCE1 stable KD cell lines constitutively expressing the shRNA targeting PIERCE1 transcript using two additional lung cancer cell lines, A549 and H460 (Supplementary Fig. 2a, b). PIERCE1 stable KD also suppressed cell proliferation (Supplementary Fig. 2c). The growth rates of both the stable KD cell lines revealed significant retardation of cell growth compared to the controls (Fig. 1c, d). Correspondingly, doxycycline-induced PIERCE1 overexpression promoted cell proliferation in A549 cells compared to controls (Fig. 1e), suggesting that PIERCE1 is required for the proliferation of most lung cancer cell lines.

**Fig. 2:**
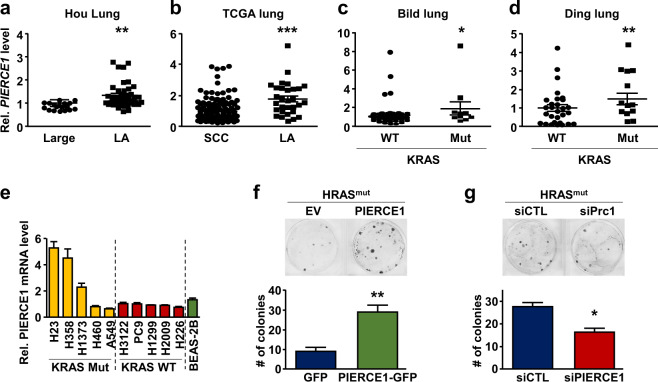
PIERCE1 expression and its tumorigenic function in KRAS-mutant lung cancer. Relative expression of *PIERCE1* transcripts in large-cell carcinoma (Large) and lung adenocarcinoma (LA) from the Hou lung database (**a**), squamous-cell carcinoma (SCC) and LA from TCGA database (**b**), KRAS wild type (WT) and mutant type (Mut) of LA from Bild lung (**c**) and Ding lung (**d**) databases, and WT and mutant KRAS lung cancer, and normal lung cell lines (**e**). KRAS genotype was indicated on the bottom of samples and cell line names. For panel **a**–**d**, Oncomine database of Hou, TCGA, Bild, and Ding were analyzed. Colony forming assays of NIH3T3 cells 14 days after transfection for overexpression (**f**) or knockdown (**g**) of PIERCE1. Colonies were formed by overexpression of mutant HRAS (HRAS^V12^). Representative images and average numbers of colonies were shown in the upper and the lower panel, respectively. Error bars are ±SD *P < 0.05; **P < 0.01; ***P < 0.0001.

Mutational spectra vary with cell types in lung cancer, which significantly affects the sensitivity and efficacy of therapies [28]. To identify the types of lung cancer cells where PIERCE1 plays a role as a tumor-promoting factor, we examined PIERCE1 expression patterns according to cellular traits and genetic mutation status. Gene expression profiles previously reported for two different NSCLC cohorts [29] revealed increased PIERCE1 expression in lung adenocarcinomas compared to large- and squamous-cell carcinomas (Fig. 2a, b). Intriguingly, the increased PIERCE1 expression in lung adenocarcinoma was found to have significant (*P* < 0.05) correlations with KRAS mutation status (Fig. 2c, d). PIERCE1 expression was higher in KRAS-mutant tumors than in WT tumors, which also appeared to be consistent in NSCLC cell lines (Fig. 2e). Moreover, an investigation of overall survival in specific types of lung cancer revealed more evident differences between low and high PIERCE1 expression groups (HR = 1.98) in lung adenocarcinomas, while no differences were detected in the case of lung squamous-cell carcinomas (Supplementary Fig. 1b, c), suggesting a link between PIERCE1 expression and survival rates particularly in lung adenocarcinomas. Notably, growths of all KRAS-mutant cells (A549, H358, H460, and H1373) were attenuated by PIERCE1 KD (Fig. 1b–d), while half of KRAS WT lung cancer cell lines (H3122, H226, HCC827, PC-9, H1299, and Heas-2B) were responsive to PIERCE1 depletion. This suggests a tumor-promoting function of PIERCE1 in KRAS-mutant lung adenocarcinomas. To confirm this hypothesis, PIERCE1 was overexpressed or knocked-down in cells expressing mutant HRAS (HRAS^V12^). PIERCE1 overexpression accelerated mutant HRAS-induced colony forming activity (Fig. 2f and Supplementary Fig. 3a), while siRNA-mediated PIERCE1 KD decreased mutant HRAS-induced colony forming activity (Fig. 2g and Supplementary Fig. 3b). In a similar manner, PIERCE1 KD decreased mutant KRAS (KRAS^G12D^)-driven colony forming activity (Supplementary Fig. 3c–e). Taken together, these data suggest that PIERCE1 is involved in the proliferation of KRAS-mutant lung cancer cells.

### PIERCE1 KD impairs mutant KRAS lung tumorigenesis through the AKT pathway

**Fig. 3:**
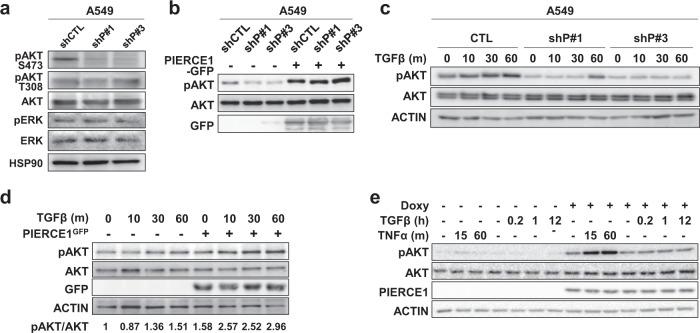
PIERCE1 promotes AKT phosphorylation in KRAS-mutant lung cancer cell lines. **a** Western blot analyses for pAKT (S473, serine 473; T308, threonine 308), AKT, pERK, and ERK in control (shCTL) and stable PIERCE1 KD (shP#1 and shP#3) A549 cell lines. HSP90 was used as a loading control. Western blot analyses for pAKT (S473) and AKT in control and stable PIERCE1 KD A549 cell lines 36 hours after transient transfection of PIERCE1-GFP (**b**) and in time dependent TGFβ-treatment conditions (**c**). GFP was used to detect overexpressed PIERCE1. ACTIN was used as a loading control. Western blot analyses for pAKT (S473), AKT under TGFβ and TNFα treatment conditions during transient transfection of PIERCE1 (**d**) or doxycycline-induced PIERCE1 overexpression condition (**e**) in A549 cells. Doxycycline (1 μg/mL) was administered for 48 hours. ACTIN was used as a loading control. Overexpression of PIERCE1 was verified using GFP or PIERCE1 antibodies.

To examine the molecular mechanism underlying PIERCE1-induced cell growth in KRAS-mutant cells, the phosphorylation status of key downstream targets of KRAS, such as AKT and ERK, were assessed in A549 cells [30]. AKT phosphorylation at S473 (pAKT) was decreased in PIERCE1 KD, although no notable changes were detected in pAKT at T308 and pERK (Fig. 3a). In these cell lines, transient overexpression of PIERCE1 rescued the PIERCE1 KD-mediated decrease in pAKT levels (Fig. 3b), indicating that PIERCE1 promotes phosphorylation of AKT at S473 residue. The level of pAKT was further examined under conditions stimulated with TGFβ, TNFα, and serum. We also found that pAKT induction was significantly reduced in PIERCE1 KD cells by the aforementioned stimuli than in the control cells (Fig. 3c and Supplementary Fig. 4a–c). As expected, PIERCE1 overexpression significantly increased pAKT (Fig. 3d, e and Supplementary Fig. 4d), suggesting that PIERCE1 promotes pAKT under various stimulus conditions.

AKT plays important roles in mutant KRAS-driven cancers and the increased expression pattern of PIERCE1 has been detected in patients with KRAS-mutant type lung cancers [31, 32]. Hence, we hypothesized that PIERCE1-mediated regulation of pAKT mainly occurs in the mutant KRAS expressing cells. To validate this hypothesis, the levels of pAKT were compared in the four KRAS-mutant cell lines (A549, H358, H23, and H1373), four KRAS wild-type (WT) lung cancer cell lines (PC-9, H1299, H3122, and H226), and normal mouse lung tissues. PIERCE1 KD reduced pAKT levels in all four KRAS-mutant cell lines (Supplementary Fig. 5a–d), while no changes were observed in KRAS WT cell lines (Supplementary Fig. 5e–h). Similar to the previous result obtained in human lung cancer cell lines, no differences were observed in normal lungs with WT KRAS of PIERCE1 TG mice, but slight down-regulation of pAKT was detected in normal lungs of PIERCE1 KO mice (Supplementary Fig. 6a, b). These data indicate that PIERCE1 enhances pAKT specifically in KRAS-mutant type of lung cancer, but not in WT KRAS. To investigate whether expression of mutant KRAS is required for PIERCE1-mediated increase of pAKT, its level was monitored in the KRAS WT and non-tumor cell lines under PIERCE1 and/or mutant KRAS overexpressed conditions. Notably, augmentation of pAKT was found in the simultaneous overexpression of PIERCE1 and mutant KRAS in KRAS WT cell lines, indicating that PIERCE1 reinforces the AKT pathway in mutant KRAS expressing condition (Supplementary Fig. 7a–c). However, pAKT was not altered by PIERCE1 overexpression alone. Regardless of PIERCE1, mutant KRAS activated MAPK/ERK signaling in all the tested cell lines and the activity for ERK was not altered further by PIERCE1 expression (Supplementary Fig. 7a–c).

**Fig. 4:**
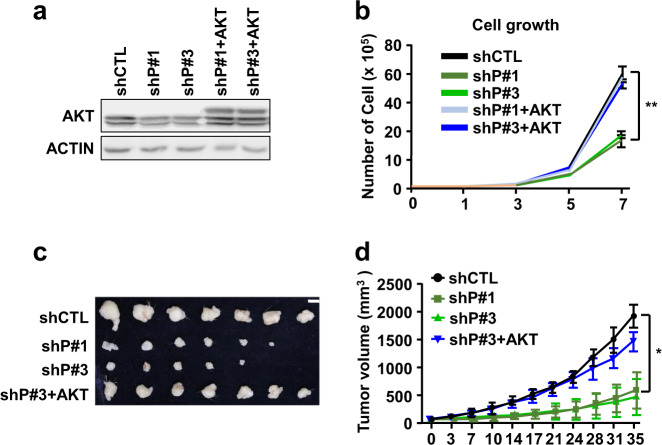
Active AKT rescues PIERCE1 KD-mediated cell growth retardation. Western blot analyses for AKT and ACTIN (**a**) and the growth rates (**b**) of control (shCTL), PIERCE1 KD (shP#1 and shP#3), and PIERCE1 KD with overexpression of myristoylated AKT (shP#1+AKT and shP#3+AKT) cell lines in A549 cell line. A representative image of xenograft tumors of cell lines 35 days after s.c. injection into nude mice (**c**) and their growth rates (**d**). White bar indicates 1 cm. Error bars represent ±SD. Statistical analysis was performed using one-way ANOVA for **b** and **d**. *P < 0.05; **P < 0.01.

AKT signaling is crucial for cell growth and survival [33], which are impaired following PIERCE1 KD, suggesting that PIERCE1 KD-mediated growth retardation might be AKT-dependent. To investigate whether the reduced cell growth rate in PIERCE1 KD cells is due to decreased pAKT levels, a myristoylated form of AKT was overexpressed in PIERCE1 KD cell lines (Fig. 4a). Analyses showed that the reduction in cell growth following PIERCE1 KD is dependent on the AKT pathway, as overexpression of its constitutively active form restored PIERCE1 KD-driven growth retardation in A549 cell lines (Fig. 4b). Moreover, by injecting these cell lines into the flank regions of nude mice in a tumor xenograft model confirmed that PIERCE1 KD-induced growth impairment could be rescued by AKT overexpression (Fig. 4c, d).

### PIERCE1 activates AKT pathway by negative regulation of TRIB3 expression

To examine which factor is involved in PIERCE1-mediated activation of AKT, we measured protein levels and activation status of various kinases and phosphatases that orchestrate AKT phosphorylation. The kinases and phosphatases involved in AKT phosphorylation at T308, such as PTEN, PI3K, PP2A, and PDK1, were not altered by PIERCE1 KD in A549 and H460 cell lines (Supplementary Fig. 8a, b) [11]. In addition, we could not find any changes in EGFR and IGF1R and their phosphorylation status (Supplementary Fig. 8c). Since mTORC2 can phosphorylate AKT at S473 [11], involvement of mTOR during PIERCE1-mediated AKT phosphorylation was inspected by suppression of key mTOR components, RAPTOR and RICTOR, which are crucial for mTORC1 and mTORC2 complex formations, respectively [34]. Compared to RAPTOR KD, PIERCE1-induced AKT phosphorylation was significantly suppressed by RICTOR KD (Supplementary Fig. 8d), indicating that mTORC2 is involved in AKT phosphorylation at S473. However, increased AKT phosphorylation was still evident in PIERCE1 overexpressing cells (Supplementary Fig. 8d). Furthermore, PIERCE1 KD did not regulate another mTORC2 downstream target PKCα (Supplementary Fig. 8e). These results suggest that alternative pathways for PIERCE1-mediated AKT phosphorylation might still exist.

**Fig. 5:**
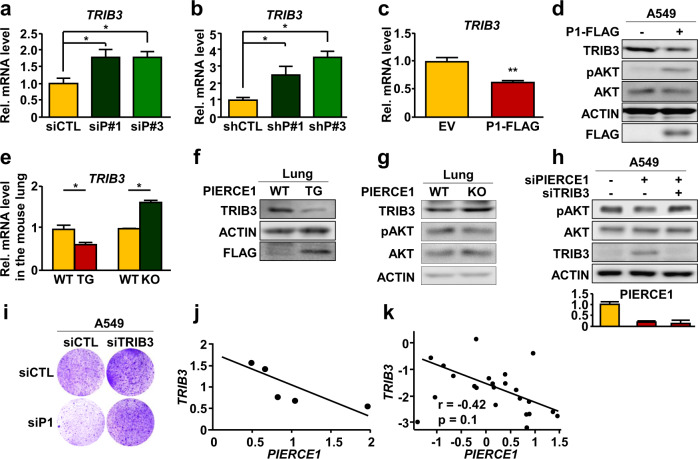
TRIB3 is required for PIERCE1-mediated activation of the AKT pathway. Relative mRNA (**a**) and protein (**b**) expression levels of TRIB3, pATK (S473), AKT, ACTIN, and FLAG 24 hours after transfection of empty vectors (EV) or PIERCE1-FLAG (P1-FLAG) in A549 cells. *ACTIN* was used as a loading control. **c**–**e** Relative mRNA levels of TRIB3 48 hours after siRNA-mediated transient KD (siP#1 and siP#3, **c**) or stable KD (shP#1 and shP#3, **d**) of PIERCE1, and in one-month old WT, PIERCE1 TG, and PIERCE1 KO mouse lungs (*n* = 3 per genotype. **e** *ACTIN* was used as a loading control (triplicated). Western blot analyses of TRIB3, actin, and FLAG in 1-month-old WT and PIERCE1 TG (**f**), and PIERCE1 KO (**g**) mouse lungs. **h** Western blot analyses for pAKT (S473), AKT, TRIB3, and ACTIN 48 hours after transient KD of indicated genes in A549 cells. **i** Monitoring of cell growth by crystal violet staining 72 hours after siRNA transfection against control (siCTL), PIERCE1 (siP1), and TRIB3 (siTRIB3) in A549 cells. **j**, **k** The ratios of *PIERCE1* and *TRIB3* expressions in lung cancer patients. Wachi Lung from Oncomine database (**j**) and The Cancer Genome Atlas (TCGA, **k**) database were used for the analyses. The r value indicates Pearson’s correlation coefficient. Error bars are ±SD *P < 0.05; **P < 0.01.

To further explore these alternative pathways, we performed gene expression analysis of A549 cells after PIERCE1 KD using three independent siRNAs against PIERCE1. We first identified 231 upregulated or 169 downregulated genes consistently by these three siRNAs (Supplementary Fig. 9a, b). The upregulated genes included two known upstream regulators of AKT, *CSK* and *TRIB3*, which might be involved in these alternative pathways. Among them, as a potential AKT regulator, we selected TRIB3, a negative regulator of AKT phosphorylation, which displayed larger expression changes by PIERCE1 KD (Supplementary Fig. 9c). Notably, TRIB3 has been known to regulate tumorigenesis specifically in the mutant HRAS expression condition [35]. In this analysis, we focused on the upregulated genes because PIERCE1 depletion reduced pAKT levels. We confirmed upregulation of TRIB3 in A549 cells after PIERCE1 KD at the mRNA level using RT-PCR (Fig. 5a, b). Correspondingly, overexpression of PIERCE1 reduced both mRNA and protein levels of TRIB3 (Fig. 5c, d). Similar mRNA and protein expression patterns of TRIB3 were observed in PIERCE1 TG and KO mouse lungs, further confirming the involvement of TRIB3 in PIERCE1-mediated AKT activation (Fig. 5e–g). As suggested by a previous study examining WT KRAS cells, the regulation of pAKT is still minimal in normal lung tissues. Therefore, we examined whether the effect of PIERCE1 against AKT activity might be compromised by alteration of TRIB3 expression in mutant KRAS cells. Accordingly, PIERCE1 KD-mediated reduction in AKT phosphorylation and cell proliferation were rescued by simultaneous KD of TRIB3 in A549 cells (Fig. 5h, i), suggesting that PIERCE1 promotes AKT phosphorylation through TRIB3. Furthermore, quantitative analysis of PIERCE1 and TRIB3 expression in lung cancer patients revealed their negative interrelationship (Fig. 5j, k), thereby confirming the correlation that PIERCE1 suppresses TRIB3 in human lung cancer patients.

Since TRIB3 expression is mainly controlled by ATF4-CHOP transcription factors that are activated upon ER stress conditions [36], we examined whether the changes in expression levels by PIERCE1 KD were enriched in CHOP target gene sets using gene set enrichment analysis (GSEA) [37]. GSEA showed significant association between PIERCE1 KD-induced expression changes with these gene sets (Supplementary Fig. 10a), suggesting the involvement of PIERCE1 in CHOP signaling. RT-PCR further confirmed that PIERCE1 KD upregulated CHOP-responsive genes, while PIERCE1 overexpression downregulated these genes (Supplementary Fig. 10b, c). Furthermore, treatment with tauroursodeoxycholic acid (TUDCA), a chemical chaperone known to reduce ER stress, could suppress PIERCE1 KD-induced activation of these genes (Supplementary Fig. 10d), suggesting that this phenomenon is ER stress-dependent. Accordingly, elimination of ER stress with TUDCA treatment inhibited PIERCE1 KD-induced TRIB3 upregulation (Supplementary Fig. 10e), suggesting that ER stress-mediated regulation of TRIB3 expression is influenced by PIERCE1 expression. PIERCE1-mediated reduction of ER stress was further confirmed by western blot analyses which showed notable decrease in TRIB3 and GRP78 expression, which is a key ER stress indicator, and induction of pAKT by transient and stable overexpression of PIERCE1 (Supplementary Fig. 11a, b), demonstrating that PIERCE1 mitigates ER stress, resulting in reduced TRIB3 expression and activation of AKT signaling. Conversely, PIERCE1 KD reinforced GRP78 and TRIB3 expressions, which were subsequently blocked by TUDCA treatment (Supplementary Fig. 11c), again supporting the theory that PIERCE1 KD promotes TRIB3 expression through ER stress. To evaluate the relevance of ER stress to cell growth retardation by PIERCE1 KD, cell proliferation was detected through crystal violet staining with or without TUDCA treatment. Approximately 60% of reduced cell growth by PIERCE1 KD was restored by TUDCA treatment (Supplementary Fig. 11d), suggesting that PIERCE1 KD-mediated protection of lung tumorigenesis occurs through induction of ER stress.

### PIERCE1 depletion suppresses lung tumorigenesis in vivo

**Fig. 6:**
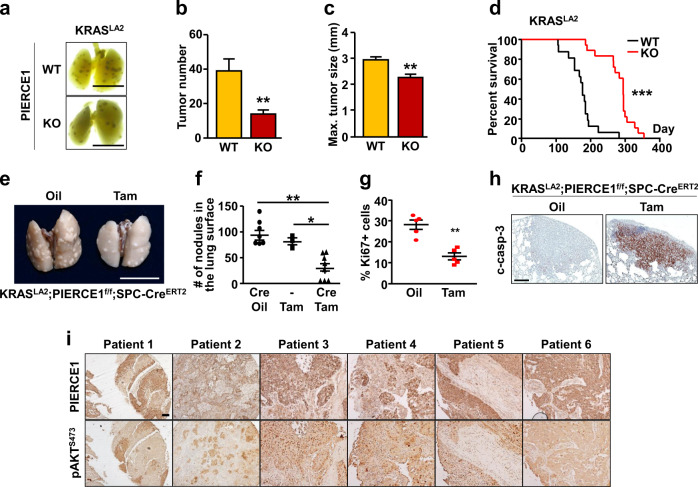
PIERCE1 KO inhibits lung tumorigenesis in vivo. **a** Representative photos of four-month-old WT and PIERCE1 KO mouse lungs in KRAS^LA2^ background. Black bars indicate 1 cm. Average numbers of tumors (**b**) and maximum tumor size (**c**) of WT (*n* = 12) and PIERCE1 KO (*n* = 12) 4-month-old mouse lungs in KRAS^LA2^ background. **d** The survival curve of WT (*n* = 16) and PIERCE1 KO (*n* = 18) mice in KRAS^LA2^ background. **e** A representative photo of oil or tamoxifen-injected mouse lungs in KRAS^LA2^;PIERCE1^f/f^;SPC-Cre^ERT2^ background. White bar indicates 1 cm. **f** Average number of tumors at the surface of lungs of oil or tamoxifen-injected mice (*n* = 8 for oil-injected Cre^Tg^, *n* = 3 for Tam-injected Cre^non-Tg^, and *n* = 8 for Tam-injected Cre^Tg^). *P < 0.05; **P < 0.01; ***P < 0.0001. Percentages of Ki67 positive cells per given field (**g**) and representative immunohistochemistry images of cleaved caspase-3 (c-casp-3, **h**) in oil- (*n* = 5) and tamoxifen-treated (*n* = 5) mouse lung tumors. Black bar indicates 100 μm. Error bars represent ±SD. Statistical analysis was performed using unpaired *t* test. **P < 0.01. **i** Immunohistochemistry analyses for PIERCE1 and pAKT (S473) expressions in human lung cancer samples. Scale bars indicate 100 μm.

To validate the clinical relevance of PIERCE1 depletion in KRAS-mutant lung cancers, we used a urethane-induced lung cancer model system that exhibits Q61R mutation in KRAS protein in 94% of all PIERCE1 KO mice by administering urethane, and these mice were examined for 3 months after initial injection [38]. Urethane injection successfully induced lung cancer as previously described [39], with an average of 20 tumor nodules in the FVB mouse strain (Supplementary Fig. 12a, b). The number and average size of urethane-induced lung tumors were significantly lower and smaller (by 27%) in PIERCE1 KO mice (Supplementary Fig. 12a–c). This observation prompted us to investigate the impact of PIERCE1 during tumorigenesis in genetic lung cancer mouse models. For this investigation, PIERCE1 KO mice were crossed with KRAS^LA2^ mice to generate mutant KRAS-induced spontaneous lung cancers [40]. Consequently, the number and size of lung tumors reduced to <50% in the 15-week-old PIERCE1 KO mice with an improved average survival rate of 50% after 185 days in WT to 290 days in KO mice (Fig. 6a–d and Supplementary Fig. 13a). This phenomenon is similar to that observed in human lung cancer patients (Fig. 1a and Supplementary Fig. 1a). Notably, the evaluation of phenotypes in PIERCE1 KO mice emphasizes the role of PIERCE1 in cancer as their survival rates and physiological activities were comparable to those of control mice (Supplementary Fig. 14), which suggest that PIERCE1 depletion had little-to-no effect during adulthood. Similar to our in vitro results, immunohistochemical analyses of the lungs of KRAS^LA2^ mice showed decreased pAKT and increased TRIB3 expression in tumor mass of PIERCE1 KO mice (Supplementary Fig. 13b), implying that ablation of PIERCE1 suppresses lung tumorigenesis with impaired AKT activity in vivo.

To test the clinical implications of PIERCE1 as a potential therapeutic strategy against lung cancer, a PIERCE1 conditional KO (cKO) mouse model was established by crossing the mice harboring the PIERCE1 KO first allele with flippase TG mice to produce a Cre-lox mouse system (Supplementary Fig. 15a) [41]. Next, PIERCE1 cKO mice were crossed with SPC (surfactant protein C)-Cre^ERT2^ knock-in (KI) mice to deplete PIERCE1 upon tamoxifen treatment (Supplementary Fig. 15a) [42]. PCR and RT-qPCR analyses were conducted to confirm lung- and time-specific deletion of PIERCE1, showing that the two doses of tamoxifen successfully caused the deletion of the PIERCE1 gene between two LoxP sites and decreased *Pierce1* transcript levels in a lung-specific manner (Supplementary Fig. 15b–d). The remaining *Pierce1* transcripts in lungs might result due to a lack of recombination events in other cell types that do not express SPC protein [43]. PIERCE1^f/f^;SPC-Cre^ERT2^ mice were crossed with KRAS^LA2^ mice to generate double (KRAS^LA2^;PIERCE1^f/f^) and triple (KRAS^LA2^;PIERCE1^f/f^;SPC-Cre^ERT2^) mutant mice. The mice harboring KRAS^LA2^ were cared for until 3 months to induce a sufficient amount of pleural lesions and tumors [40], and they were treated with tamoxifen to examine the effect of PIERCE1 elimination before and during lung tumorigenesis, and they were sacrificed 2 months after the first injection of tamoxifen (Supplementary Fig. 15e). Gross morphological analyses revealed that the average number of lung tumors in 5-month-old KRAS^LA2^ mice was ~93, while 30 tumor nodules were detected in tamoxifen-treated mice (Fig. 6e, f), which might be due to the reduced proliferation and increased tumor cell death (Fig. 6g, h). Tamoxifen alone did not affect tumor burden without Cre (Fig. 6f). To further confirm our findings, an allograft of a mutant KRAS expressing lung cancer cell line (LL/2) was subcutaneously introduced into the flank regions of C57BL6/J mice and siRNA against PIERCE1 was administered into tumor mass every three days (Supplementary Fig. 16a). Correspondingly, tumor volume and weight were suppressed by up to 70% by siRNA-mediated PIERCE1 KD (Supplementary Fig. 16b–e). Collectively, these data suggest that PIERCE1 inhibition provides clinical benefit particularly for mutant KRAS-driven lung cancer.

Expressional analyses in tissue microarray of human lung cancer specimens were performed to investigate PIERCE1 and pAKT levels. Based on PIERCE1 expression levels in normal and lung cancer tissues, NSCLC samples were split into three groups, namely, low *PIERCE1* expression (expression levels similar to those of normal lung tissues), middle (expression degree 10–40%), and high (expression degree higher than 40%) (Supplementary Fig. 17). The middle and high PIERCE1 expression groups were counted as PIERCE1-positive lung cancer samples which account for 83.4% of the cases, suggesting that PIERCE1 is expressed in most lung cancer patients. Importantly, of those middle and high PIERCE1 expression specimens, 75% of tumor samples showed co-expression patterns with pAKT, with only two exceptional cases (Fig. 6i), thereby suggesting that PIERCE1 expression is linked to the activation of the AKT pathway in human lung cancer. Taken together, these findings from human, mouse, and in vitro experiments are consistent with our hypothesis.

## Discussion

**Fig. 7:**
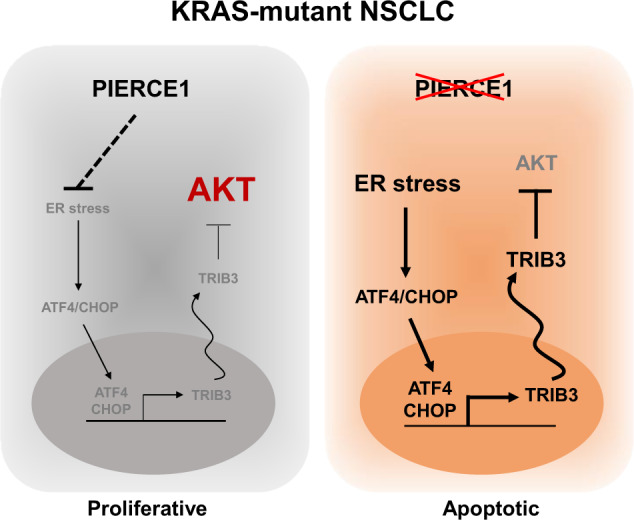
Representative model of PIERCE1 in lung cancer. A schematic representation of PIERCE1 in KRAS-mutant lung cancer, and its role in the AKT pathway. PIERCE1 ablation induces ER stress-mediated TRIB3 expression, resulting in inhibition of the AKT pathway and apoptosis.

Here, we explored the underlying mechanisms of action of PIERCE1 in KRAS-mutant lung cancer, which accounts for 30% of all lung adenocarcinoma cases [7]. PIERCE1 activates PI3K/AKT pathway, which is one of the major downstream signaling pathways of KRAS implicated in tumorigenesis [10]. Intervention of PIERCE and AKT in ER stress-induced TRIB3 expression [20] shows that PIERCE1 modulates AKT phosphorylation in mutant KRAS-driven lung cancer. Moreover, we showed that inhibition of PIERCE1 expression before and after the onset of tumorigenesis effectively hindered cancer cell growth. Thus, this study will provide a clinical benefit to KRAS-mutant lung cancer studies (Fig. 7).

PIERCE1 depletion successfully suppressed tumor cell growth and decreased AKT phosphorylation, specifically in KRAS-mutant lung cancer cells. Notably, TRIB3 controls tumorigenesis of HRAS- or PTEN-mutant cells [18, 35], which shows that PIERCE1-mediated regulation of tumorigenesis is dependent on oncogenic RAS and TRIB3. In addition, the tumor suppressive function of PIERCE1 is not tightly limited to mutant KRAS expression because it also hinders cell growth in WT KRAS expressing cells. In that case, as pAKT levels were not altered by PIERCE1 KD, its molecular mechanism needs to be further studied. Although both HCC827 and PC-9 cell lines harbor EGFR mutations, PIERCE1 KD suppressed proliferation only in HCC827, suggesting the existence of other factors that determine responsiveness to PIERCE1 KD.

Functional study of pancreatic cancer has shown that stimulation of ER stress under the mutant KRAS expressing condition, which has higher sensitivity to stimulants for ER stress, such as tunicamycin, brefeldin A, and bortezomib, results in an anticancer effect in mutant KRAS expressing conditions [44]. Moreover, unresolved ER stress induced by a high-calorie diet reduces the tumorigenic potential of mutant KRAS-driven lung cancer [25], which suggests that ER stress is a potential drug discovery target for KRAS-mutant lung cancer [45]. In our study, PIERCE1 KD promoted ER stress responsive genes, such as ATF3 and CHOP, negatively regulating AKT phosphorylation, a key contributor toward ER stress-induced cell death and hindrance in cell proliferation [46, 47]. Suppression of ER stress by TUDCA substantiates our conclusion that PIERCE1 depletion inhibits AKT phosphorylation indirectly by reducing ER stress.

MAPK and AKT pathways govern crucial physiological processes, such as proliferation and survival, in KRAS-mutant lung cancer [32]. Inhibition of the MAPK pathway alone is insufficient to cure KRAS-mutant lung cancer because, as a monotherapy, it causes drug resistance through enhanced AKT signaling [48]. Hence, simultaneous inhibition of both MAPK and AKT pathways might be a more effective anticancer therapy to improve clinical outcomes. Several clinical trials have investigated the inhibition of the PI3K/AKT/mTOR pathway with small molecule inhibitors, and although these molecules improved the progression-free survival of patients, they were toxic [49, 50]. The toxicity observed in clinical trials could be explained by phenotypes such as embryonic lethality and growth impairment in KO mouse models [51–53]. The essential function of the PI3K/AKT/mTOR pathway and its expression in almost all tissues necessitate the development of novel drugs targeting this pathway [54]. This study reveals that the antitumorigenic effect of PIERCE1 depletion is controlled by the AKT pathway in lung cancer, while ERK activity is not affected by PIERCE1. Complete depletion of PIERCE1 in mice inhibits AKT phosphorylation in tumors, which promotes cell death and induces apoptosis [55], while no notable adverse effects were detected in the current study, suggesting a novel drug candidate that influences the AKT pathway with minimal side effects. Although the complete KO of PIERCE1 does not affect mouse phenotype after birth and no evident differences in AKT phosphorylation were detected in normal tissues, this may be due to the partial embryonic lethality caused by incomplete *situs inversus* (heterotaxy) [3]. Therefore, the small molecules targeting PIERCE1 might show no significant adverse effects in adults probably towing to its limited expression in specific tissues such as the lungs, brain, and kidneys [1], even though it can be harmful during embryo development. Tumor allograft studies also suggest the potential of an anti-PIERCE1 lung cancer therapy. Although KD efficiency should be improved as only a 50% decrease in PIERCE1 expression was observed, siRNA-mediated PIERCE1 KD suppressed tumor growth in KRAS-mutant lung cancer. Overall, we discovered that the mechanism underlying PIERCE1 KD-mediated suppression in KRAS-mutant lung tumorigenesis is driven by the TRIB3-AKT axis. Our study elucidates the effects of PIERCE1 KD and provides evidence to suggest that PIERCE1 might be a novel therapeutic target for KRAS-mutant NSCLC.

## Materials and methods

### Reagents and plasmids

Doxycycline, puromycin, and crystal violet were purchased from Sigma-Aldrich (USA). Human PIERCE1 cDNA was subcloned into the following vectors: pCW57-RFP-P2A-MCS (Addgene, Cambridge, MA, USA) for doxycycline inducible plasmid, pCS4-3×flag for Flag-tagging, pEGFP-C1 for GFP-tagging. pLNCX-myr-HA-AKT1 plasmid was purchased from Addgene (USA) and used to generate stable cell lines. Lentiviral vectors for shPIERCE1 expression were purchased from Sigma-Aldrich (USA). siRNAs for control, PIERCE1, RICTOR, RAPTOR, and TRIB3 were purchased from GenePharma. Lipofectamine 3000 (Invitrogen, USA), Lipofectamine RNAiMAX (Invitrogen, USA), and jetPEI (Polyplus-transfection SA, France) were used for transfection.

### Cell culture, cell proliferation assay, and generation of stable cell lines

A549 and H1299 cell lines were purchased from ATCC (Manassas, VA, USA). H460, H358, H23, H226, PC-9, NIH3T3, HCC827, and H3122 cell lines were purchased from Korean Cell Line Bank (Seoul, Korea). HACAT and BEAS-2B cell lines were generous gifts from Dr. Hyun Woo Park (Department of Biochemistry, Yonsei University, Seoul, Korea). The cell lines were maintained in DMEM and RPMI (HyClone, USA) media supplemented with penicillin/streptomycin (Gibco, Carlsbad, CA, USA) and 10% of FBS (HyClone, USA) at 37 °C with 5% CO_2_ in a humidified chamber. For proliferation assay, 2 × 10^4^ cells were seeded onto 60 mm dishes.

### RNA purification and Real-time quantitative PCR analysis

RNA purification and cDNA synthesis were performed as described previously [56]. Briefly, total RNA was isolated using TRIzol reagent (Ambion, Life technologies, USA), following which cDNA was synthesized using a RevertAid First strand cDNA Synthesis Kit (Thermo Fisher Scientific Inc, USA). Real-time quantitative PCR (RT-qPCR) was conducted using SensiFASTTM Sybr No-Rox Mix (Bioline, Australia). Quantitative gene expression analyses were performed using a CFX384 Real-Time PCR system (Bio-Rad, Berkeley, CA, USA). qPCR was performed with the primers indicated in the Supplementary Table and purchased from Origene Technologies, Inc. (USA).

### Statistical analyses

Statistical significance was determined using the two-tailed *t* test. Data were analyzed with GraphPad Prism (GraphPad Software Inc, USA). *P* values < 0.05 were considered statistically significant.

## Supplementary information

Supplementary Information

Supplementary Figure

Supplementary Table
