# Supplementary material for: Impaired AKT signaling and lung tumorigenesis by PIERCE1 ablation in KRAS-mutant non-small cell lung cancer
Source: Oncogene. 2020 Jul 29;39(36):5876–87. doi: 10.1038/s41388-020-01399-5 (PMC7471098; doi:10.1038/s41388-020-01399-5)
Supplement: Supplementary file 1 — Supplementary Information [file 41388_2020_1399_MOESM1_ESM.docx]

**Supplementary Information**

**Impaired AKT signaling and lung tumorigenesis by PIERCE1 ablation**

**in KRAS-mutant non-small cell lung cancer**

Jae-il Roh^1*^, Jaehoon Lee^1*^, Young-Hoon Sung^2^, Jahyun Oh^1^, Do Young Hyeon^3^, Yujin Kim^1^, Seungeon Lee^1^, Sushil Devkota^4^, Hye Jeong Kim^1^, Bomin Park^1^, Taewook Nam^1^, Yaechan Song^1^, Yonghwan Kim^5^, Daehee Hwang^3^, and Han-Woong Lee^1,#^

**Supplementary Materials and Methods**

**Colony forming assay**

NIT-3T3 cells were seeded at a density of 1 × 10^6^ onto 60-mm dishes and were transfected with HRAS^G12V^ or KRAS^G12D^ together with PIERCE1-GFP or its control-GFP vectors, or transfected with siRNA against PIERCE1 or control by Lipofectamine 3000 and Lipofectamine RNAiMAX reagents (Invitrogen), respectively. Two days after transfection, 1 × 10^5^ cells were split into three 35-mm dishes and treated with G418 (400 μg/mL). Two weeks after selection, cells were stained with crystal violet and colonies were counted.

**Gene set enrichment analysis**

Gene set enrichment analysis was performed using GSEA v3.0 software [1]. Gene sets BIOCARTA_AKT_PATHWAY and BIOCARTA_ERK_PATHWAY obtained from GSEA website were used for enrichment of AKT and ERK signaling pathway-related genes, respectively. The gene set used for enriching CHOP target genes was obtained from a previous study [2]. The gene sets with nominal *p-*value less than 0.05 computed from the GSEA software were selected as the ones enriched by the comparison methods used.

**Immunoblotting**

Immunoblotting was performed as described previously [3]. Antibodies against AKT (#9272), pAKT (#9275), PP2A B (#2290), PP2A C (#2038), pPDK1 (#3061), RICTOR (#2114), RAPTOR (#2280), SIN-1 (#12860), GβL (#3227), PI3K (#4292), PTEN (#9188), EGFR (#4267), p-EGFR (#3777), IGFR1 (#9750), pIGFR1 (#4568), and GRP78 (#3177) were purchased from Cell Signaling Technologies. Antibodies against Pan-RAS (sc-166691), ACTIN (sc-47778), GAPDH (sc-47724), GFP (sc-9996), TRIP3 (sc-165964), and HSP90 (sc-13119) were purchased from Santa Cruz Biotechnology, Inc. FLAG (F3165) antibody was purchased from Sigma-Aldrich Inc. Human PIERCE1 fragments were synthesized and injected into the rabbit for polyclonal antibody production (Bethyl Laboratories).

**Kaplan-Meier plotter database analysis**

A Kaplan-Meier Plotter survival analysis containing the gene expression data and survival information of patients with lung cancer was used for analyzing overall survival, progression-free survival, hazard ratio, and *p*-value [4]. Probe #59437_at was used for analyses. The patients were grouped according to the auto selection of the best cut-off scores.

**Microarray** **analysis**

Total RNA was isolated from A549 cells transfected with one of the three different sequences of siPIERCE1 (siP1 #1, siP1 #2, and siP1 #3) or control siRNA (siControl). The integrity of the total RNA was measured in all the samples using a Bioanalyzer 2100 (Agilent, Santa Clara, CA, USA) and was confirmed to be sufficient for gene expression analysis (RNA integrity number > 9). RNA was reverse-transcribed and amplified, and then hybridized onto the array (Agilent-039494 SurePrint G3 Human GE v2 8x60k), which includes 62,976 probes corresponding to 23,705 annotated genes following the standard Agilent protocols. The mRNA levels were measured for two biological replicates in each condition. The gene expression data were deposited to the Gene Expression Omnibus database (GSE136131). The probe intensities measured from the microarray experiments were converted to log_2_-intensities and were normalized using quantile normalization. We first identified the expressed genes as previously described [5]. Among the expressed genes, we then identified differentially expressed genes (DEGs) for the following comparisons using an integrative statistical method previously reported [5]: 1) siP1 #1 vs siControl; 2) siP1 #2 vs siControl; and 3) siP1 #3 vs siControl. Briefly, for each gene, we calculated a T-statistic value using Student’s t-test and a log_2_-median-ratio in each comparison. We then estimated empirical distributions of T-statistic values and log_2_-median-ratios for the null hypothesis (i.e. the genes are not differentially expressed) by random permutation experiments of all samples. Using the estimated empirical distributions for each gene, we computed adjusted *p*-values for the observed T-statistic value and log_2_-median-ratio and then calculated the overall *p*-value by combining these *p*-values using Stouffer’s method [6]. Finally, we identified DEGs as the genes that have overall *p*-values < 0.05 and absolute log_2_-median-ratios > 0.58 (1.5-fold).

**Hematoxylin and Eosin (H&E) staining and immunohistochemistry (IHC)**

Lung tissues were fixed in neutral-buffered formalin and embedded in paraffin blocks according to the standard procedure, and sections were stained with hematoxylin and eosin (H&E) as described previously [7]. Lung carcinoma tissue microarray was purchased (LC1501 and LUC1021, US Biomax, Inc.) for the analyses. For immunohistochemistry, slices were rehydrated, incubated with citrate buffer for antigen retrieval, and the manufacturer’s instructions were followed (#13079, Cell Signaling Technologies) for the remaining reactions. Cleaved caspase 3 antibody (#9661) was purchased from Cell Signaling Technologies and Ki-67 (NB110-89717) was purchased from Novus Biologicals.

**Mouse models, Urethane-induced lung tumorigenesis, xenograft, and allograft models**

PIERCE1 KO mice were established as described previously [8]. KRAS^LA2^ mouse was purchased from the Jackson Laboratory and crossed with PIERCE1 KO mice to maintain the C57BL/6 strain. Mice were maintained as described previously [9]. Briefly, the mice were fed a normal diet (PicoLab® Rodent Diet 20, Orient Bio, Inc.) under a 12-hour light/dark cycle in a specific pathogen-free (SPF) facility of the Yonsei Laboratory Animal Research Center. KRAS^LA2^;PIERCE1^+/+^ and KRAS^LA2^;PIERCE1^-/-^ mice were sacrificed and the lungs were analyzed at 15 weeks of age. ES cells harboring the PIERCE1 KO first allele (1700007K13Rik^tm1a(KOMP)Wtsi^) were obtained from the Knockout Mouse Project (KOMP) and used for the generation of PIERCE1 cKO mouse model by crossing with ACTN-Flp TG mice (Stock #003800 from Jackson Laboratories). KRAS^LA2^;PIERCE1^f/f^ mice were crossed with SPC-Cre^ERT2^ Tg that were kindly provided by Dr. Brigid L. M. Hogan (Duke University) [10] to produce KRAS^LA2^;PIERCE1^f/f^ and KRAS^LA2^;PIERCE1^f/f^;SPC-Cre^ERT2^. Tamoxifen (4 mg/day) was administered to twelve-week old mice four times every other day. Mice were sacrificed 2 months after the first injection. Urethane (1 g/kg) was randomly administered to 1-month-old mice in a FVB background four times weekly through i.p. injection. Mice were sacrificed and lung cancers were analyzed 3 months after the first injection. Five-week-old female BALB/c-nu Slc mice were purchased from Central Lab. Animal Inc. (Korea) for performing xenograft assays. After anesthesia by avertin, 1 × 10^7^ cells were transplanted into the flanks of mice. Genotyping was performed with the following primers: PIERCE1 cKO-Common, 5’-CGA AGG CCA ATT AGT GAA GTC AAG C-3’; PIERCE1 cKO-WT, 5’-CCA GAG AAC AGG ACT AAG AAG CAC G-3’; PIERCE1 cKO-KO, 5’-ATA AAC CCT CTT GCA GTT GCA TC-3’; PIERCE1 KO-general-F, 5’-CGA AGG CCA ATT AGT GAA GTC AAG C-3’; PIERCE1 KO WT-R, 5’-CCA GAG AAC AGG ACT AAG AAG CAC G-3’; PIERCE1 KO-R, 5’-ATA AAC CCT CTT GCA GTT GCA TC-3’; PIERCE1 TG-F, 5’-GCA ACG TGC TTG TTA TTG TG-3’; PIERCE1 TG-R, 5’-AAT ATC ACG GGT AGC GAA CG-3’; Cre F, 5’-GTC GAT GCA ACG AGT GAT GA-3’; Cre-R, 5’-TCA TCA GCT ACA CCA GAG AC-3’; KRAS^LA2^ Common-F, 5’-TGC ACA GCT TAG TGA GAC CC-3’; KRAS^LA2^ WT-R, 5’-GAC TGC TCT CTT TCA CCT CC-3’; and KRAS^LA2^ Mut-R, 5’-GGA GCA AAG CTG CTA TTG GC-3’. LL/2 cells were harvested, counted, prepared at a density of 2 × 10^6^ cells per 100 μL supplemented with Matrigel matrix (Corning, #354234), and randomly injected into the flanks of female C57BL6/J mice. Prior to the injection, the mice were anesthetized with Avertin (Sigma Aldrich). One day after tumor injection, siRNA was mixed with jetPEI (siRNA, 5 μg; jetPEI, 0.8 μL) according to the manufacturer’s instructions and administered into the tumor mass. Tumor volume was measured every three days before injection and calculated based on the following equation: Volume = (tumor length × tumor width^2^)/2. The mouse genotypes were blinded during tumor size measurements. Animal experimental procedures were conducted in accordance with the Korean Food and Drug Administration (KFDA) guidelines, and approved by the Institutional Animal Care and Use Committees (IACUC) of Yonsei University (Permit Number: IACIC-A-201610-435-02).

**Confirmation of Cre-induced PIERCE1 depletion**

Fifteen week-old PIERCE1^f/f^;SPC-Cre^ERT2^ mice were administered with either only oil or tamoxifen (4 mg/mouse) in oil twice and sacrificed the next day. Genomic DNA was isolated from mouse tails and organs as described previously [7]. PCR was performed with the following primers: Forward, 5’-ACA AGT TTG TAC AAA AAA GCA GGC T-3’; and Reverse, 5’-TTG ACT TCA CTA ATT GGC CTT CG-3’.

**Supplementary Figure legends**

**Supplementary Fig. 1. Probable involvement of PIERCE1 in human lung cancers**

**a-c** Kaplan-Meier plotter analyses of progression-free survival (PFS) of 982 lung cancer patients (a), and overall survival (OS) in 710 lung adenocarcinoma patients (**b**), and 524 squamous cell carcinoma (SCC) patients (**c**) with respect to PIERCE1 expression low (black) and high (red) groups. Auto select best cutoff was chosen for the analyses. Probe 59437_at was used for analyzing PIERCE1 levels. The hazard ratio (HR) and *p*-values are indicated. Statistical analysis was performed by log-rank test.

**Supplementary Fig. 2. Production of stable PIERCE1 KD cell lines using shRNAs against PIERCE1**

**a, b** Relative mRNA levels of *PIERCE1* compared to controls (shCTL) in the stable PIERCE1 KD A549 (shP1#1 and shP1#3) and stable PIERCE1 KD H460 cell lines (shP#2 and shP#3). **c** Representative crystal violet stained images indicating colony formation of stable PIERCE1 KD A549 cells on day 6.

**Supplementary Fig. 3. Impaired mutant KRAS-induced colony forming activity by PIERCE1 KD in NIH3T3 cells**

**a, b** Western blot analyses for RAS, GFP and Actin proteins 48 h after transfection of PIERCE1-GFP or siRNA against PIERCE1 and HRAS^V12^ in NIH3T3 cells. Actin was used as a loading control. **c, d** Representative photos of KRAS^G12D^-induced colony formation in NIH3T3 cells (**c**) and its quantification (**d**). **e** Relative *Pierce1* expression levels at 48 h after siRNA transfection against control (CTL) or PIERCE1 (Prc1) in NIH3T3 cells (triplicates). Error bars represent ± SD. Statistical analysis was performed using unpaired *t*-test. **P* < 0.05.

**Supplementary Fig. 4. Impaired stimulant-induced AKT phosphorylation using PIERCE1 KD**

**a-c** Western blot analyses for pAKT (S473), AKT in TGFβ- (**a**), TNFα- (**b**), and serum- (**c**) treatment conditions in the control (shCTL), and PIERCE1 KD (shP#1 and shP#3) A549 cell lines. HSP90 and ACTIN were used as loading controls. **d** Western blot analyses for pAKT (S473) under TNFα-treatment conditions in PIERCE1 overexpressed A549 cells. **e** Western blot analyses for pAKT (S473), AKT, and HSP90 in the control (shCTL) and PIERCE1 KD (shP#2 and shP#3) H460 cell lines.

**Supplementary Fig. 5. PIERCE1 promotes AKT phosphorylation in KRAS-mutant lung cancer cell lines**

**a–h** Western blot analyses for pAKT (S473) and AKT proteins at 48 h after siRNA transfection against the control (siCTL) and PIERCE1 (siP#1 and siP#3) in the mutant KRAS cells A549 (**a**), H358 (**b**), H23 (**c)**, H1373 (**d**), and the WT KRAS cells PC-9 (**e**), H1299 (**f)**, H3122 (**g**), and H226 (**h**). ACTIN was used as a loading control. Relative mRNA levels of *PIERCE1* compared to controls (shCTL) in each cell line are indicated in the lower panel of each western blotting dataset. Error bars are ± SD.

**Supplementary Fig. 6. AKT phosphorylation in the normal lung tissues of PIERCE1 TG and KO mice**

**a, b** Western blot analyses for pAKT (S473) and AKT proteins in the lungs of PIERCE1 TG (**a**) and KO (**b**) mouse compared to WT. Flag was used to confirm Flag-PIERCE1 overexpression in TG mice. Actin was used as a loading control.

**Supplementary Fig. 7. PIERCE1 promotes AKT phosphorylation in the mutant KRAS expression condition**

**a–c** Western blot analyses for pAKT (S473), AKT, PIERCE1, RAS, HSP90, ERK, and pERK in H226 (**a**), H3122 (**b**), and RPE-1 (**c**) cell lines 24 h after transfection of indicated constructs.

**Supplementary Fig. 8. Kinases and phosphatases related to AKT phosphorylation at T308 are not altered by PIERCE1**

**a, b** Western blot analyses for PTEN, pPHLPP, PI3K, PP2As, pPDK1, and HSP90 in the control (shCTL) and PIERCE1 KD (shP#1 and shP#3) A549 (**a**) and H460 (shP#2 and shP#3) (**b**) cell lines. **c** Western blot analyses for EGFR, pEGFR, IGF1R, pIGF1R, and HSP90 in the control (shCTL) and PIERCE1 KD (shP#1 and shP#3) A549 cell lines. **d** Western blot analyses for pAKT (S473), AKT, RAPTOR, and RICTOR 24 h after siRNA transfection. Indicated amount of doxycycline was treated for 2 days to induce PIERCE1 expression. **e** Western blot analyses for pPKCα, PKCα, pAKT, AKT, and ACTIN in the control (shCTL) and PIERCE1 KD (shP#1 and shP#3) A549 cell lines.

**Supplementary Fig. 9. Identification of AKT upstream regulators involved in the alternative signaling pathways through gene expression analysis**

**a, b** Venn diagrams showing relationships between up-regulated (**a**) and down-regulated genes (**b**) in A549 cells by three independent siRNAs against PIERCE1 (siP#1, siP#2, and siP#3). The red boxes indicate 231 and 169 genes up- (**a**) and down- (**b**) regulated consistently by the three independent siRNAs. **c** Log_2_-fold-changes of two known upstream AKT regulators upregulated by PIERCE1 KD. Positive values imply upregulation in PIERCE1 KD condition, compared to that in the control.

**Supplementary Fig. 10. PIERCE1 suppresses ES stress-induced TRIB3 activation in A549 cells**

**a** Gene set enrichment analyses (GSEA) plots in three independent PIERCE1-siRNA treated cells for CHOP target genes (normalized enrichment score = 1.373 in siP#1, 1.23 in siP#2, and 1.525 in siP#3). Nominal *P*-value = 0 for all the plots. siP1, siRNA for PIERCE1; siCTL, siRNA for control. **b, c** Relative levels of transcripts of indicated genes in the control (siCTL, black color) and PIERCE1 KD (siP#1 in yellow, and siP#3 in orange color) (**b**), or MOCK (empty vector, black color) and PIERCE1 (brown color) overexpressed (**c**) A549 cells. *ACTIN* was used as a loading control. **d, e** Relative transcript levels of indicated genes in the control (siCTL, black color) and PIERCE1 KD (siP#1 in yellow, and siP#3 in orange color). TUDCA was treated for 48 h. Error bars represent ± SD. Statistical analysis was performed using unpaired *t*-test. **P* < 0.05, ***P* < 0.01, ns indicates not significant.

**Supplementary Fig. 11. Control of ER stress status links PIERCE1-mediated activation of AKT pathway**

**a–c** Western blot analyses for GRP78, FLAG, TRIB3, ACTIN, and GAPDH 24 h after transient overexpression of PIERCE1 in a dose dependent manner (- indicates no expression, + indicates low dose, +++ indicates high dose of PIERCE1 transfection, **a**), or control (MOCK) and PIERCE1 stably overexpressing cell lines (P#1 and P#2, **b**), or 48 h after siRNA transfection against control (CTL) or PIERCE1 (#1 and #3, **c**) in A549 cells. **d** Crystal violet staining (upper) and its relative intensities (lower) in A549 cells 72 h after siRNA transfection against control (siCTL) and PIERCE1 (siP#1 and siP#3). TUDCA was treated for 48 h (**c**) or 72 h (**d**). Error bars represent ± SD. Statistical analysis was performed using unpaired *t*-test. ***P* < 0.01, ns indicates not significant.

**Supplementary Fig. 12. PIERCE1 KO inhibits urethane-induced lung tumorigenesis *in vivo***

**a** Representative photos of the lungs of mice at 3 months after urethane injection in WT and PIERCE1 KO mice. Black bars indicate 1 cm. **b, c** Average numbers of tumors (**b**) and maximum tumor size (**c**) in the WT (n = 7) and PIERCE1 KO (n = 9) mouse lungs 3 months after urethane injection.

**Supplementary Fig. 13. Immunohistochemistry of PIERCE1 KO mouse lungs in KRAS^LA2^ background**

**a** Representative photos of H&E staining of the lung tissues of 4-month old WT and PIERCE1 KO mice in KRAS^LA2^ background. Black bar indicates 100 μm. **b** Representative immunohistochemistry images for pAKT (S473) and TRIB3 in the 4-month-old KRAS^LA2^-induced lung tumors of WT and PIERCE1 KO mice. Black bars indicate 100 μm.

**Supplementary Fig. 14. PIERCE1 depletion does not affect mouse survival rates**

Kaplan-Meier’s survival plot of WT (black, *n* = 25) and PIERCE1 KO (red, *n* = 43) mice.

**Supplementary Fig. 15. Tamoxifen-driven selective deletion of PIERCE1 in the PIERCE1 cKO mice**

**a** A schematic map of genomic DNA of WT and PIERCE1 cKO (tm1) mice. LacZ and neo selection markers are inserted between exons 1 and 2 with two FRP sites (red semicircle) and one LoxP site (yellow arrowhead), and exon 2 is flanked by LoxP sites. LacZ and neo markers were eliminated by crossing with Flp TG mice (tm1c). Crossing of Pierce1<tm1c> with Cre recombinase TG mice results in deletion of exon 2 (tm1d, Δ/Δ). Blue arrows indicate PCR primers used for panels **b** and **c**. (**b**) PCR analysis of genomic DNA for WT mouse tail, and PIERCE1 cKO mouse tail and lung 3 days after TAM injection. **c** PCR analyses of genomic DNA for indicated tissues in the indicated genetic backgrounds with or without tamoxifen treatment. Tamoxifen or oil was administered twice. **d** Relative *Pierce1* transcript levels in the indicated tissues after oil (yellow) or tamoxifen (dark red) treatment. Bra, brain; Hrt, heart; Liv, Liver; Kid, kidney; Spl, spleen; Int, intestine. *Gapdh* was used as a loading control. **e** Scheme indicating tamoxifen injection and analysis of lung tumors in mice. Tamoxifen or oil was administered in 3-month-old mice four times; their lungs were analyzed 2 months after the first injection.

**Supplementary Fig. 16. siRNA-mediated PIERCE1 KD suppresses mutant KRAS lung cancer growth in allograft model**

**a** Scheme indicating tumor (LL/2 cell line) and siRNA injection strategy. siRNA against control (CTL) or PIERCE1 (Prc1) was administered every three days for eight times. **b** KD efficiency of PIERCE1 in the tumor mass 10 days after tumor injection. **c** A representative photo of tumor mass 22 days after allograft. White bar indicates 1 cm. **d, e** Growth rate of allograft tumor mass at the indicated date (**d**) and tumor weight 22 days after first injection (**e**) in the siRNA for control (siCTL, n = 7) and PIERCE1 (siPrc1, n = 7) administration condition. Error bars represent ± SD. Statistical analysis was performed using unpaired *t*-test. **P* < 0.05.

**Supplementary Fig. 17. Expression pattern of PIERCE1 in human lung cancer patients**

Immunohistochemistry scoring for PIERCE1 expression levels in normal and lung cancer patient samples. Scale bars indicate 100 μm.

**Supplementary references**

1. Subramanian A, Tamayo P, Mootha VK, Mukherjee S, Ebert BL, Gillette MA, et al. Gene set enrichment analysis: a knowledge-based approach for interpreting genome-wide expression profiles. Proc Natl Acad Sci U S A. 2005;102(43):15545-50.

2. Yang Y, Liu L, Naik I, Braunstein Z, Zhong J, Ren B. Transcription Factor C/EBP Homologous Protein in Health and Diseases. Front Immunol. 2017;8:1612.

3. Roh JI, Kim Y, Oh J, Kim Y, Lee J, Lee J, et al. Hexokinase 2 is a molecular bridge linking telomerase and autophagy. PLoS One. 2018;13(2):e0193182.

4. Nagy A, Lanczky A, Menyhart O, Gyorffy B. Validation of miRNA prognostic power in hepatocellular carcinoma using expression data of independent datasets. Sci Rep. 2018;8(1):9227.

5. Chae S, Ahn BY, Byun K, Cho YM, Yu MH, Lee B, et al. A systems approach for decoding mitochondrial retrograde signaling pathways. Sci Signal. 2013;6(264):rs4.

6. Hwang D, Rust AG, Ramsey S, Smith JJ, Leslie DM, Weston AD, et al. A data integration methodology for systems biology. Proc Natl Acad Sci U S A. 2005;102(48):17296-301.

7. Roh JI, Cheong C, Sung YH, Lee J, Oh J, Lee BS, et al. Perturbation of NCOA6 leads to dilated cardiomyopathy. Cell Rep. 2014;8(4):991-8.

8. Sung YH, Baek IJ, Kim YH, Gho YS, Oh SP, Lee YJ, et al. PIERCE1 is critical for specification of left-right asymmetry in mice. Sci Rep. 2016;6:27932.

9. Roh JI, Lee J, Park SU, Kang YS, Lee J, Oh AR, et al. CRISPR-Cas9-mediated generation of obese and diabetic mouse models. Exp Anim. 2018;67(2):229-37.

10. Rock JR, Barkauskas CE, Cronce MJ, Xue Y, Harris JR, Liang J, et al. Multiple stromal populations contribute to pulmonary fibrosis without evidence for epithelial to mesenchymal transition. Proc Natl Acad Sci U S A. 2011;108(52):E1475-83.
