# Supplementary material for: Impaired AKT signaling and lung tumorigenesis by PIERCE1 ablation in KRAS-mutant non-small cell lung cancer
Source: Oncogene. 2020 Jul 29;39(36):5876–87. doi: 10.1038/s41388-020-01399-5 (PMC7471098; doi:10.1038/s41388-020-01399-5)
Supplement: Supplementary file 2 — Supplementary Figure [file 41388_2020_1399_MOESM2_ESM.pptx]

## Slide 1
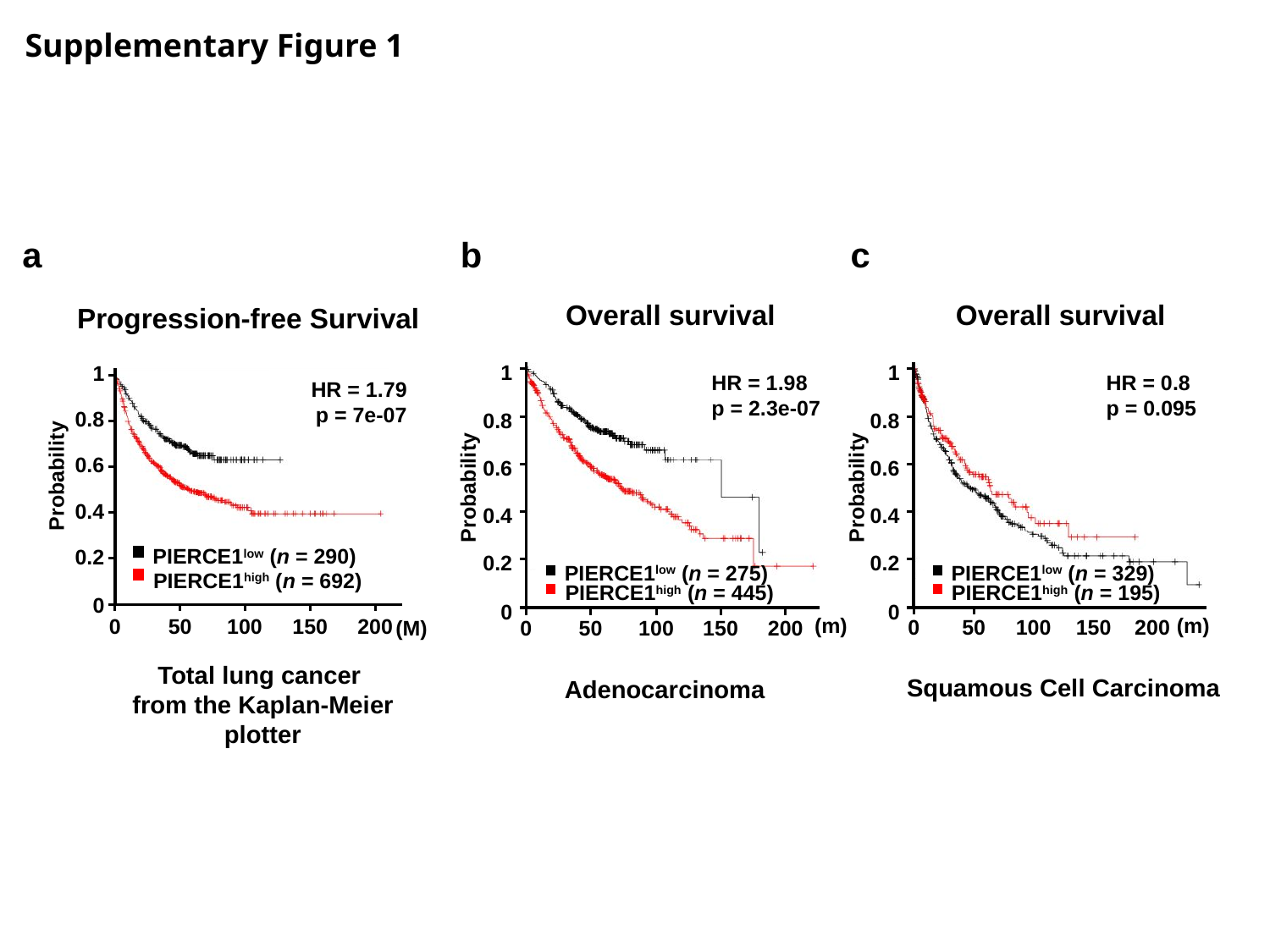

Supplementary Figure 1
a
b
c
Overall survival
1
1
HR = 1.98
p = 2.3e-07
HR = 0.8
p = 0.095
0.8
0.8
0.6
0.6
Probability
Probability
0.4
0.4
0.2
0.2
PIERCE1low (n = 275)
PIERCE1low (n = 329)
PIERCE1high (n = 445)
PIERCE1high (n = 195)
0
0
(m)
(m)
0
50
100
150
200
0
50
100
150
200
Squamous Cell Carcinoma
Adenocarcinoma
Overall survival
Progression-free Survival
1
HR = 1.79
p = 7e-07
0.8
0.6
Probability
0.4
PIERCE1low (n = 290)
0.2
PIERCE1high (n = 692)
0
0
50
100
150
200
(M)
Total lung cancer
from the Kaplan-Meier plotter

## Slide 2
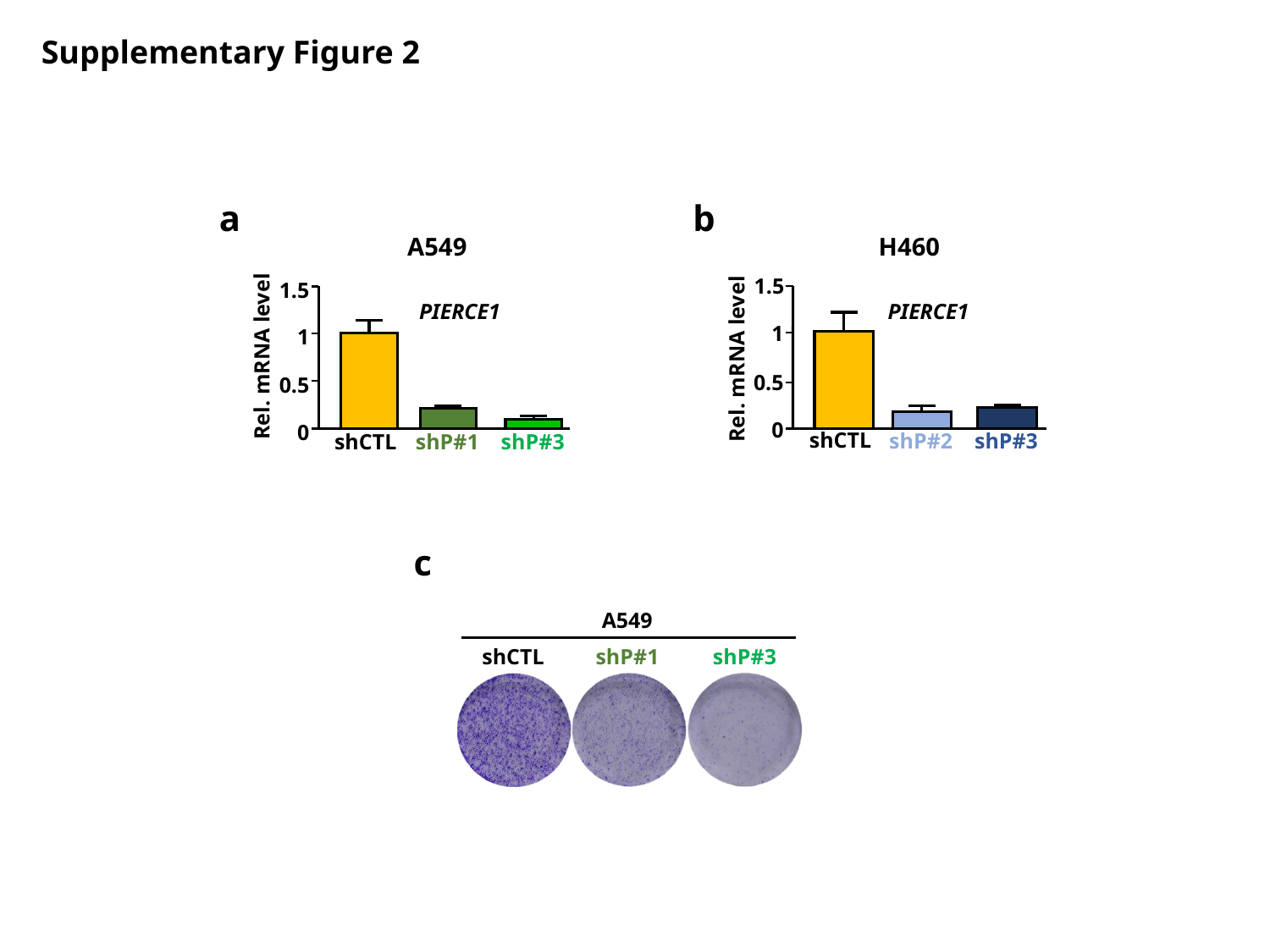

Supplementary Figure 2
a
b
A549
H460
1.5
1.5
PIERCE1
PIERCE1
1
1
Rel. mRNA level
Rel. mRNA level
0.5
0.5
0
0
shCTL
shP#2
shP#3
shCTL
shP#1
shP#3
c
A549
shCTL
shP#1
shP#3

## Slide 3
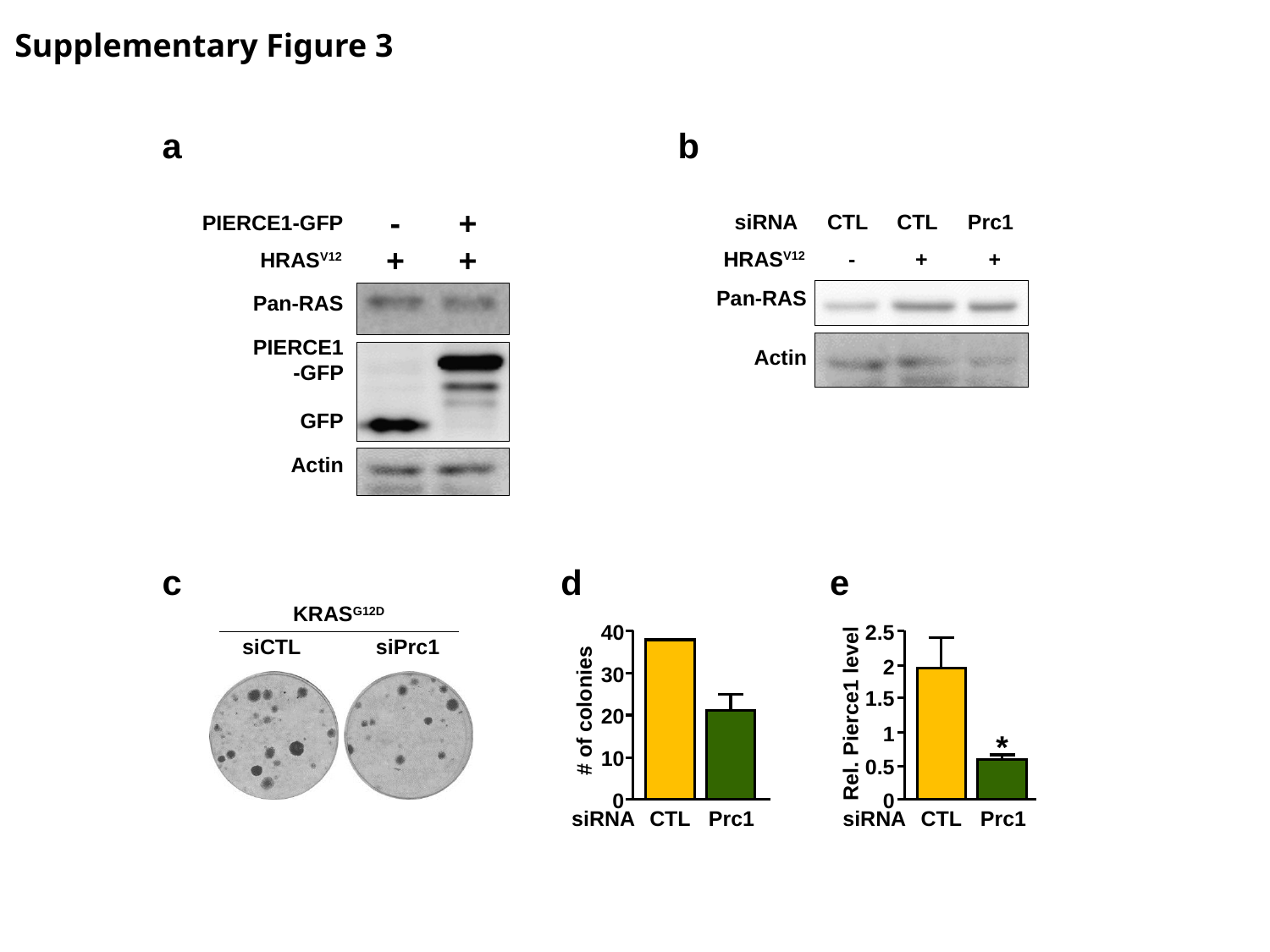

Supplementary Figure 3
a
b
-
+
siRNA
CTL
CTL
Prc1
PIERCE1-GFP
+
+
HRASV12
-
+
+
HRASV12
Pan-RAS
Pan-RAS
PIERCE1
-GFP
Actin
GFP
Actin
c
d
e
KRASG12D
40
2.5
siCTL
siPrc1
2
30
1.5
# of colonies
Rel. Pierce1 level
20
1
*
10
0.5
0
0
siRNA
CTL
Prc1
siRNA
CTL
Prc1

## Slide 4
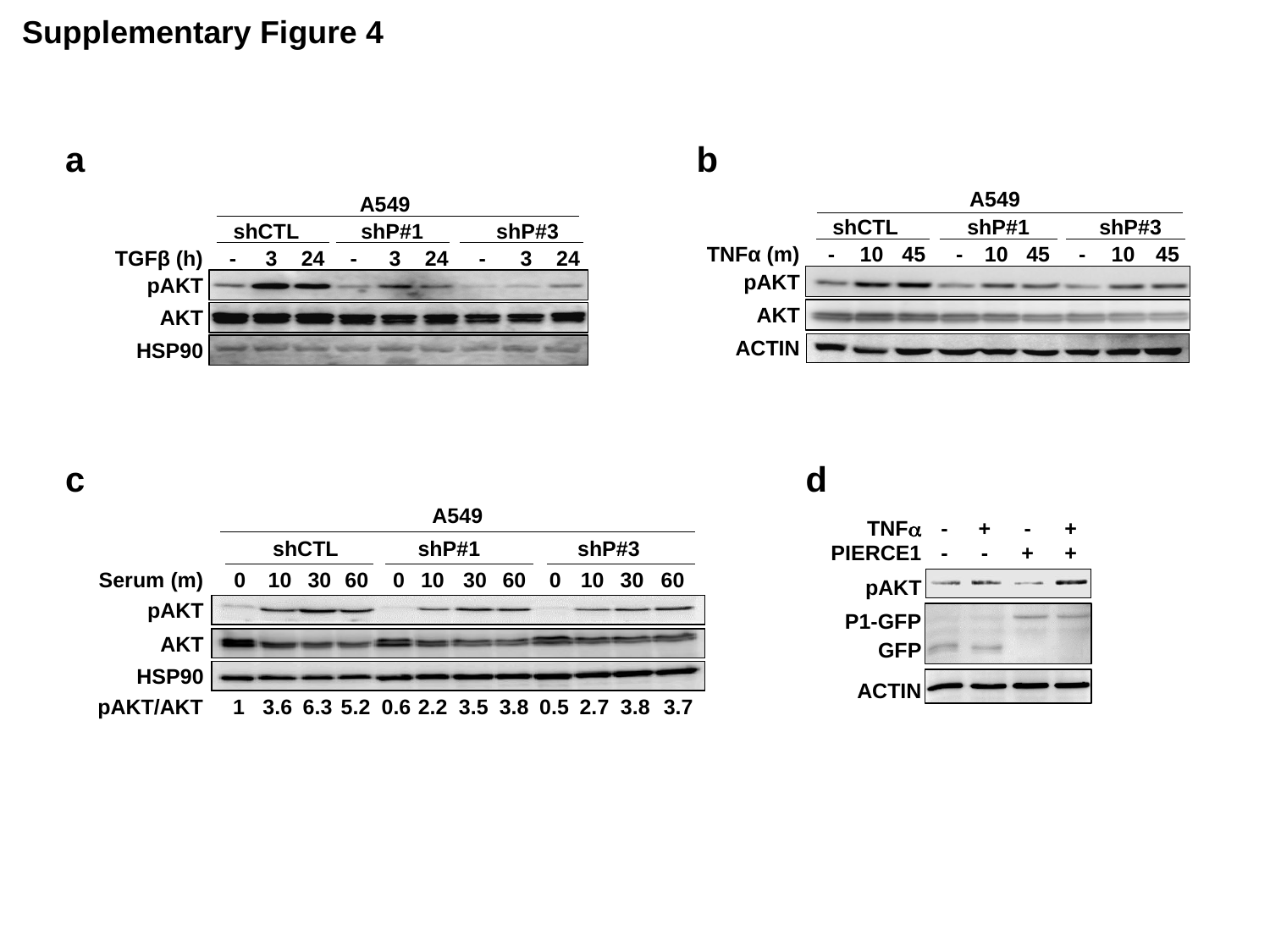

Supplementary Figure 4
a
b
A549
A549
shCTL
shP#1
shP#3
TGFβ (h)
-
3
24
-
3
24
-
3
24
pAKT
AKT
HSP90
shCTL
shP#1
shP#3
TNFα (m)
-
10
45
-
10
45
-
10
45
pAKT
AKT
ACTIN
c
d
A549
shCTL
shP#1
shP#3
Serum (m)
0
10
30
60
0
10
30
60
0
10
30
60
pAKT
AKT
HSP90
pAKT/AKT
1
3.6
6.3
5.2
0.6
2.2
3.5
3.8
0.5
2.7
3.8
3.7
TNFα
-
+
-
+
PIERCE1
-
-
+
+
pAKT
P1-GFP
GFP
ACTIN

## Slide 5
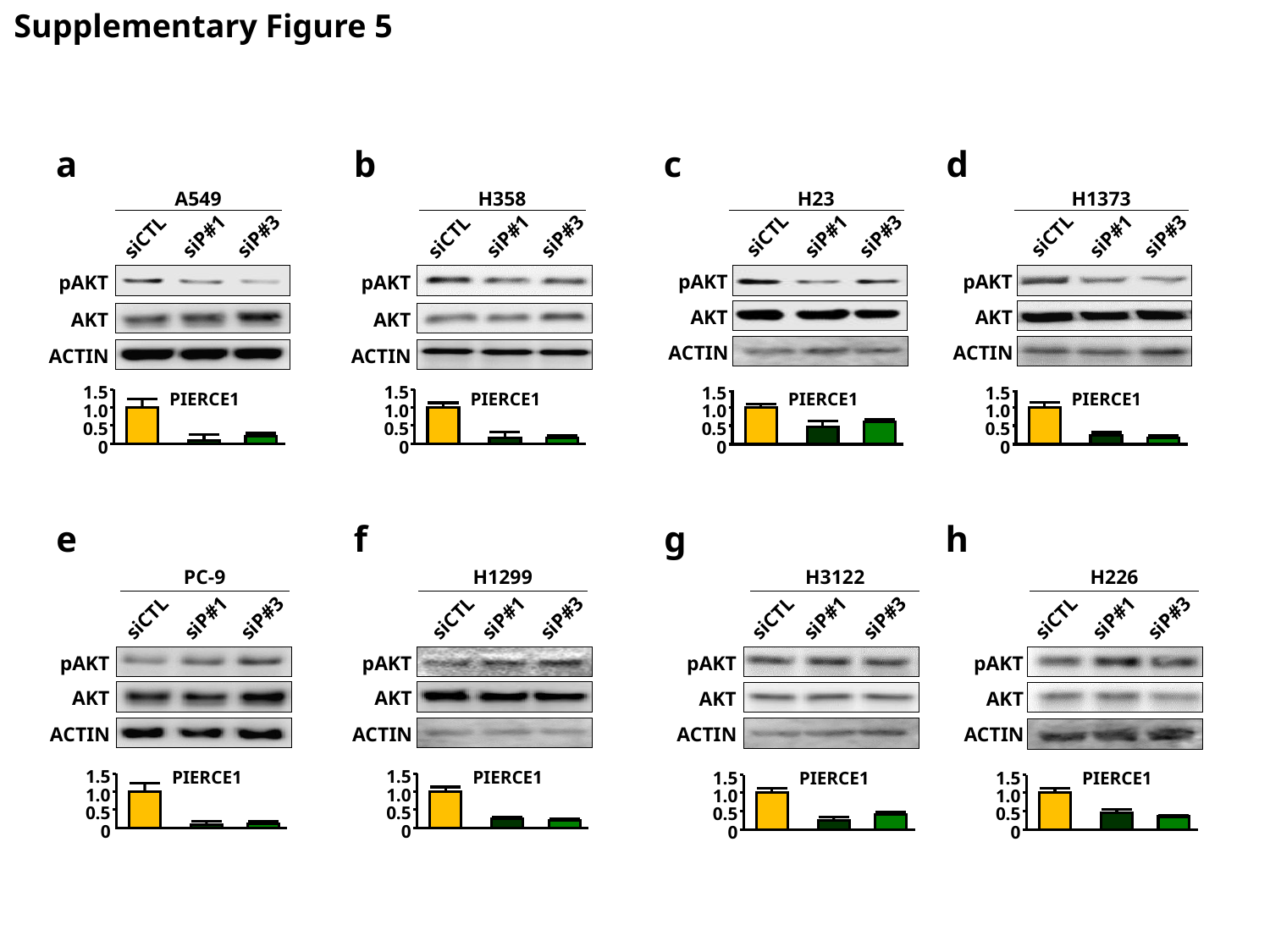

Supplementary Figure 5
a
b
c
d
A549
H358
H23
H1373
siCTL
siCTL
siP#1
siP#3
siP#1
siP#3
siP#1
siP#3
siP#1
siP#3
siCTL
siCTL
pAKT
pAKT
pAKT
pAKT
AKT
AKT
AKT
AKT
ACTIN
ACTIN
ACTIN
ACTIN
1.5
1.5
1.5
1.5
PIERCE1
PIERCE1
PIERCE1
PIERCE1
1.0
1.0
1.0
1.0
0.5
0.5
0.5
0.5
0
0
0
0
e
f
g
h
PC-9
H1299
H3122
H226
siCTL
siP#1
siP#3
siCTL
siP#1
siP#3
siCTL
siP#1
siP#3
siCTL
siP#1
siP#3
pAKT
pAKT
pAKT
pAKT
AKT
AKT
AKT
AKT
ACTIN
ACTIN
ACTIN
ACTIN
1.5
1.5
PIERCE1
PIERCE1
1.5
1.5
PIERCE1
PIERCE1
1.0
1.0
1.0
1.0
0.5
0.5
0.5
0.5
0
0
0
0

## Slide 6
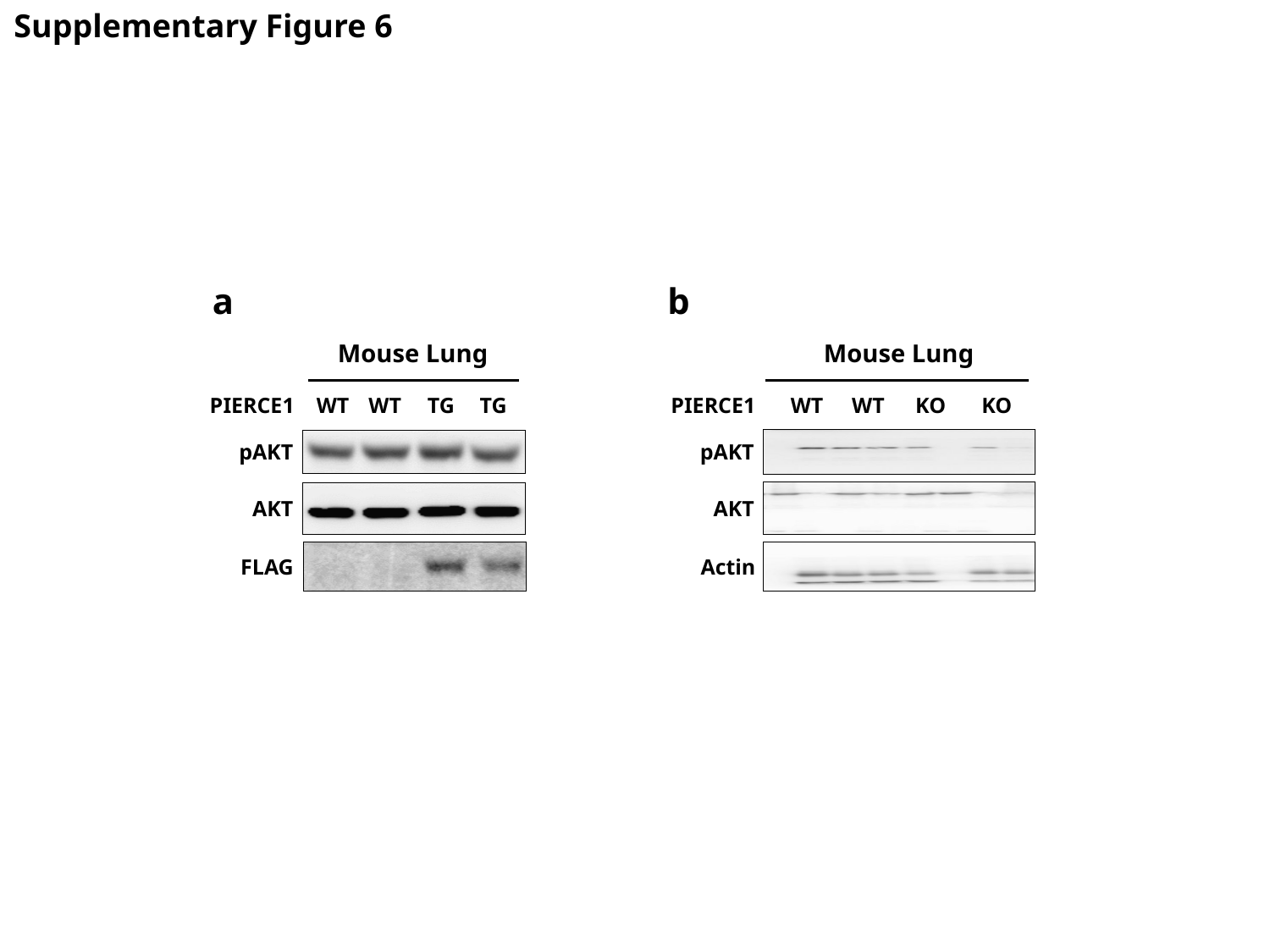

Supplementary Figure 6
a
b
Mouse Lung
Mouse Lung
WT
WT
KO
KO
PIERCE1
WT
WT
TG
TG
PIERCE1
pAKT
pAKT
AKT
AKT
FLAG
Actin

## Slide 7
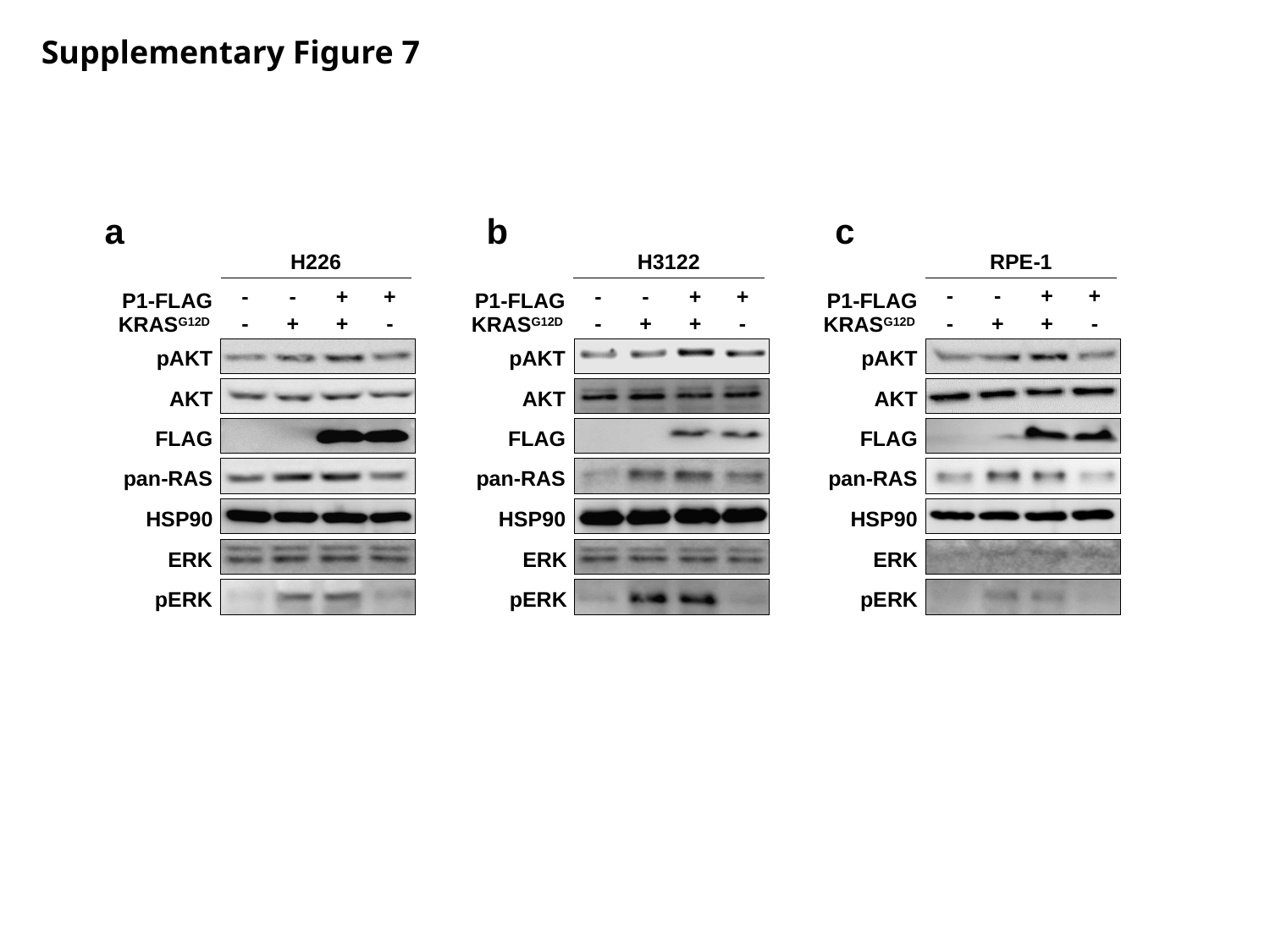

Supplementary Figure 7
a
b
c
RPE-1
H226
H3122
-
-
+
+
-
-
+
+
-
-
+
+
P1-FLAG
P1-FLAG
P1-FLAG
-
+
+
-
-
+
+
-
-
+
+
-
KRASG12D
KRASG12D
KRASG12D
pAKT
pAKT
pAKT
AKT
AKT
AKT
FLAG
FLAG
FLAG
pan-RAS
pan-RAS
pan-RAS
HSP90
HSP90
HSP90
ERK
ERK
ERK
pERK
pERK
pERK

## Slide 8
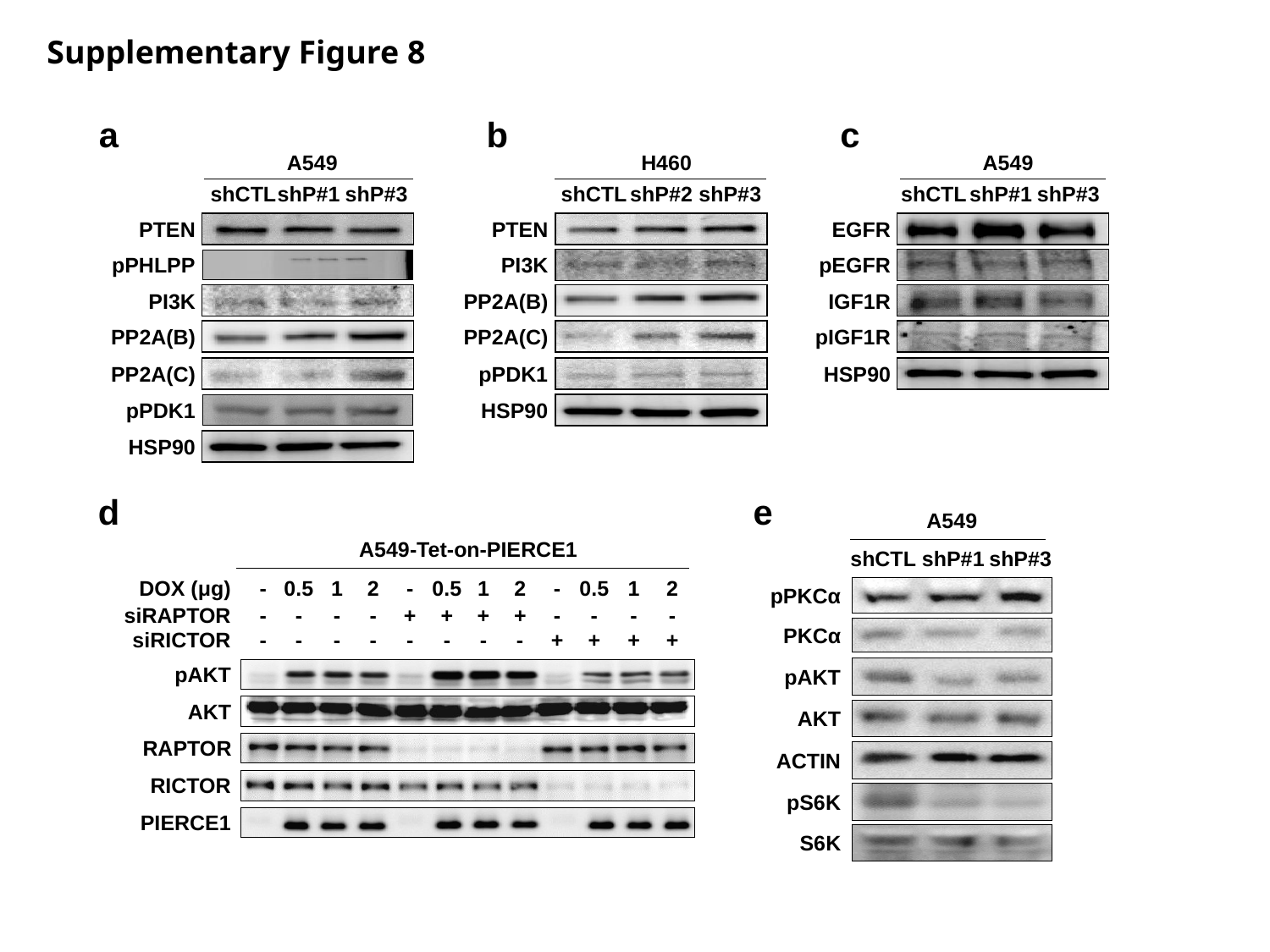

Supplementary Figure 8
a
b
c
A549
H460
A549
shCTL
shP#1
shP#3
shCTL
shP#2
shP#3
shCTL
shP#1
shP#3
PTEN
PTEN
EGFR
pPHLPP
PI3K
pEGFR
PI3K
PP2A(B)
IGF1R
PP2A(B)
PP2A(C)
pIGF1R
PP2A(C)
pPDK1
HSP90
pPDK1
HSP90
HSP90
d
e
A549
A549-Tet-on-PIERCE1
shCTL
shP#1
shP#3
DOX (μg)
-
0.5
1
2
-
0.5
1
2
-
0.5
1
2
pPKCα
siRAPTOR
-
-
-
-
+
+
+
+
-
-
-
-
PKCα
siRICTOR
-
-
-
-
-
-
-
-
+
+
+
+
pAKT
pAKT
AKT
AKT
RAPTOR
ACTIN
RICTOR
pS6K
PIERCE1
S6K

## Slide 9
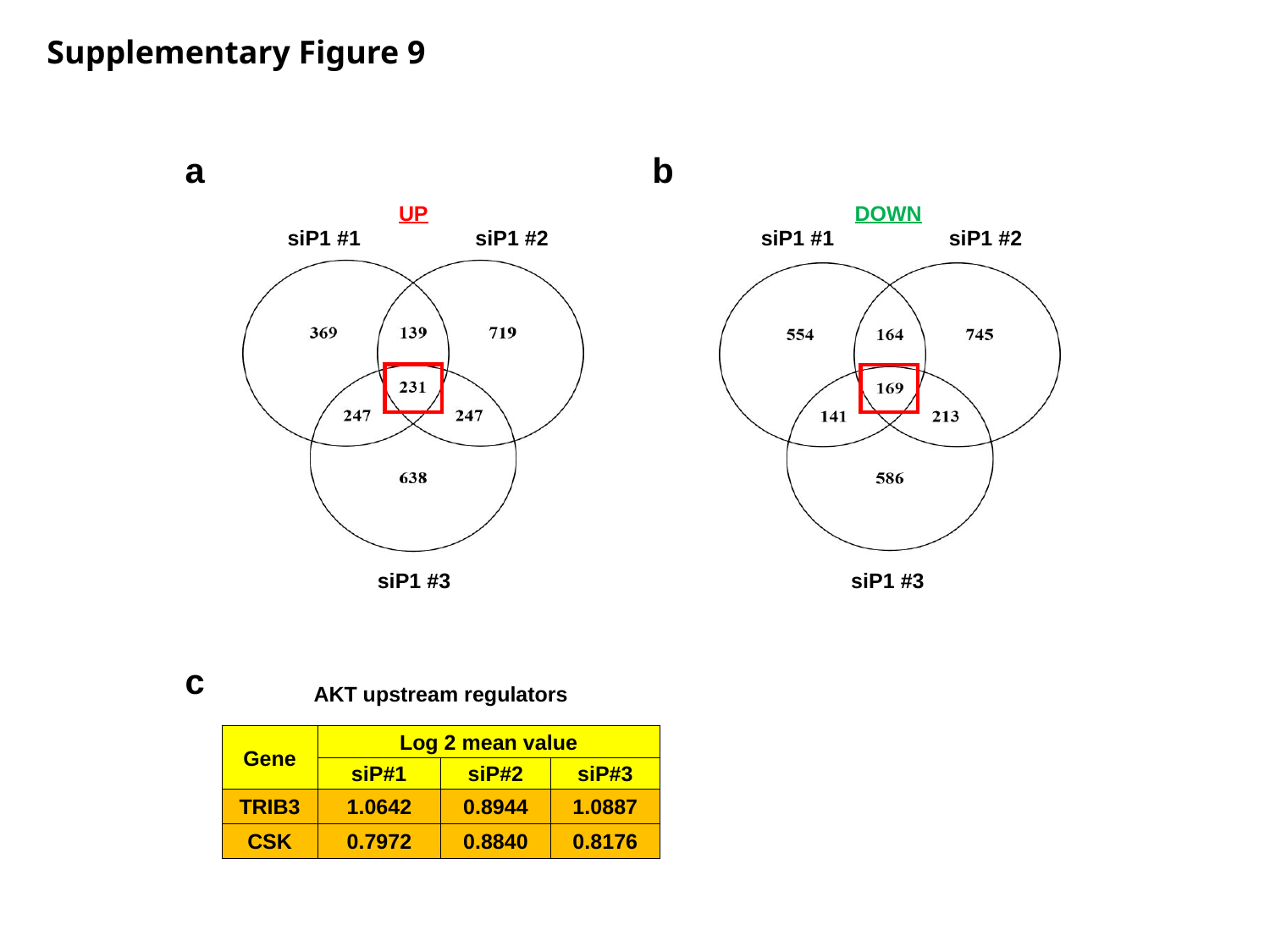

Supplementary Figure 9
a
b
UP
DOWN
siP1 #1
siP1 #2
siP1 #1
siP1 #2
siP1 #3
siP1 #3
c
AKT upstream regulators
| Gene | Log 2 mean value | | |
| --- | --- | --- | --- |
| | siP#1 | siP#2 | siP#3 |
| TRIB3 | 1.0642 | 0.8944 | 1.0887 |
| CSK | 0.7972 | 0.8840 | 0.8176 |

## Slide 10
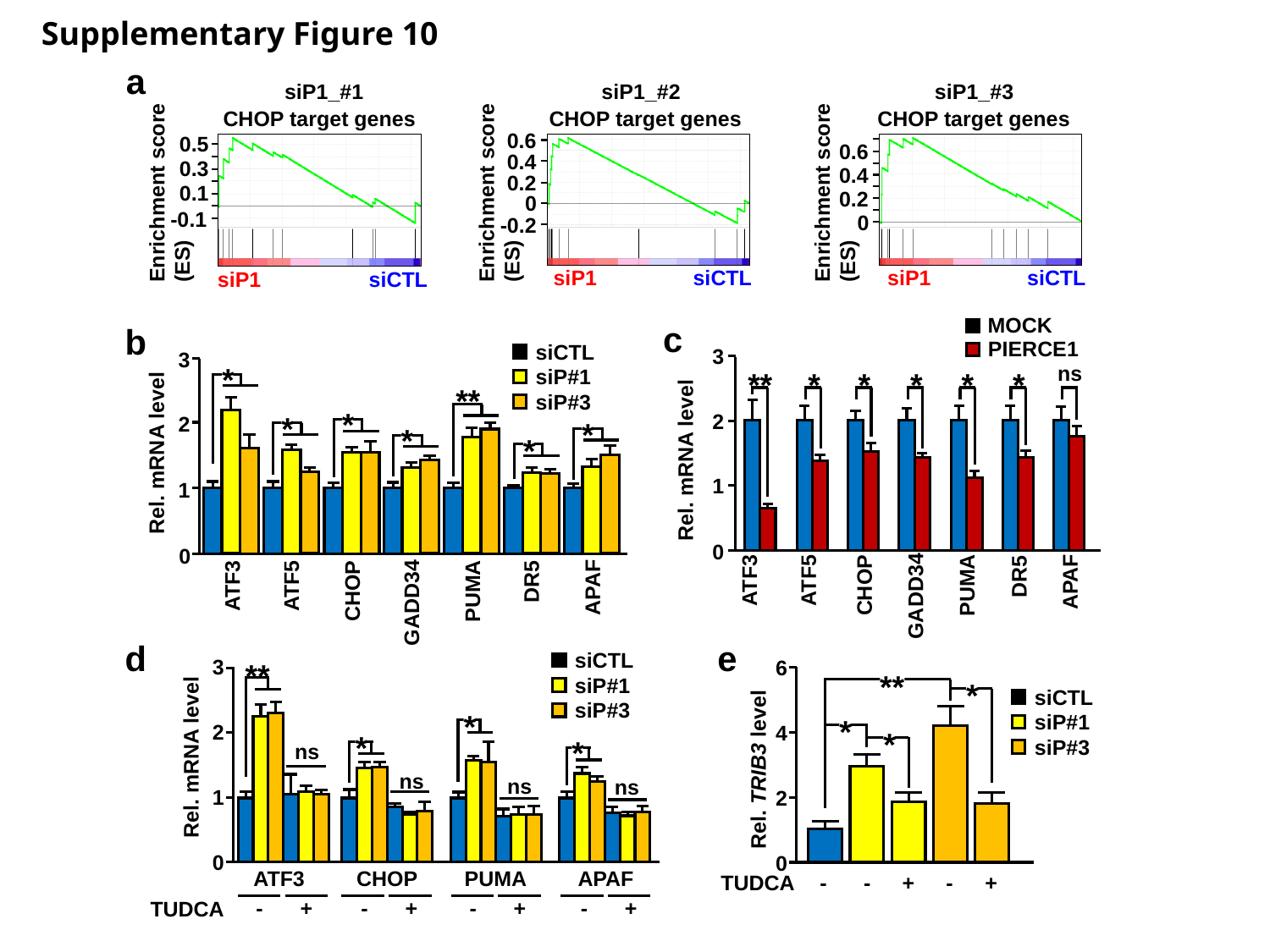

Supplementary Figure 10
a
siP1_#1
CHOP target genes
0.5
0.3
Enrichment score
(ES)
0.1
-0.1
siP1
siCTL
siP1_#2
CHOP target genes
0.6
0.4
Enrichment score
(ES)
0.2
0
-0.2
siP1
siCTL
siP1_#3
CHOP target genes
0.6
Enrichment score
(ES)
0.4
0.2
0
siP1
siCTL
c
MOCK
b
siCTL
3
*
siP#1
**
siP#3
*
*
2
*
*
*
Rel. mRNA level
1
0
DR5
ATF3
ATF5
APAF
CHOP
PUMA
GADD34
PIERCE1
3
ns
**
*
*
*
*
*
2
Rel. mRNA level
1
0
DR5
ATF3
ATF5
APAF
CHOP
PUMA
GADD34
d
e
siCTL
3
**
siP#1
siP#3
*
2
*
*
ns
Rel. mRNA level
ns
ns
ns
1
0
ATF3
CHOP
PUMA
APAF
-
+
-
+
-
+
-
+
TUDCA
6
**
*
siCTL
siP#1
*
4
*
siP#3
Rel. TRIB3 level
2
0
TUDCA
-
-
+
-
+

## Slide 11
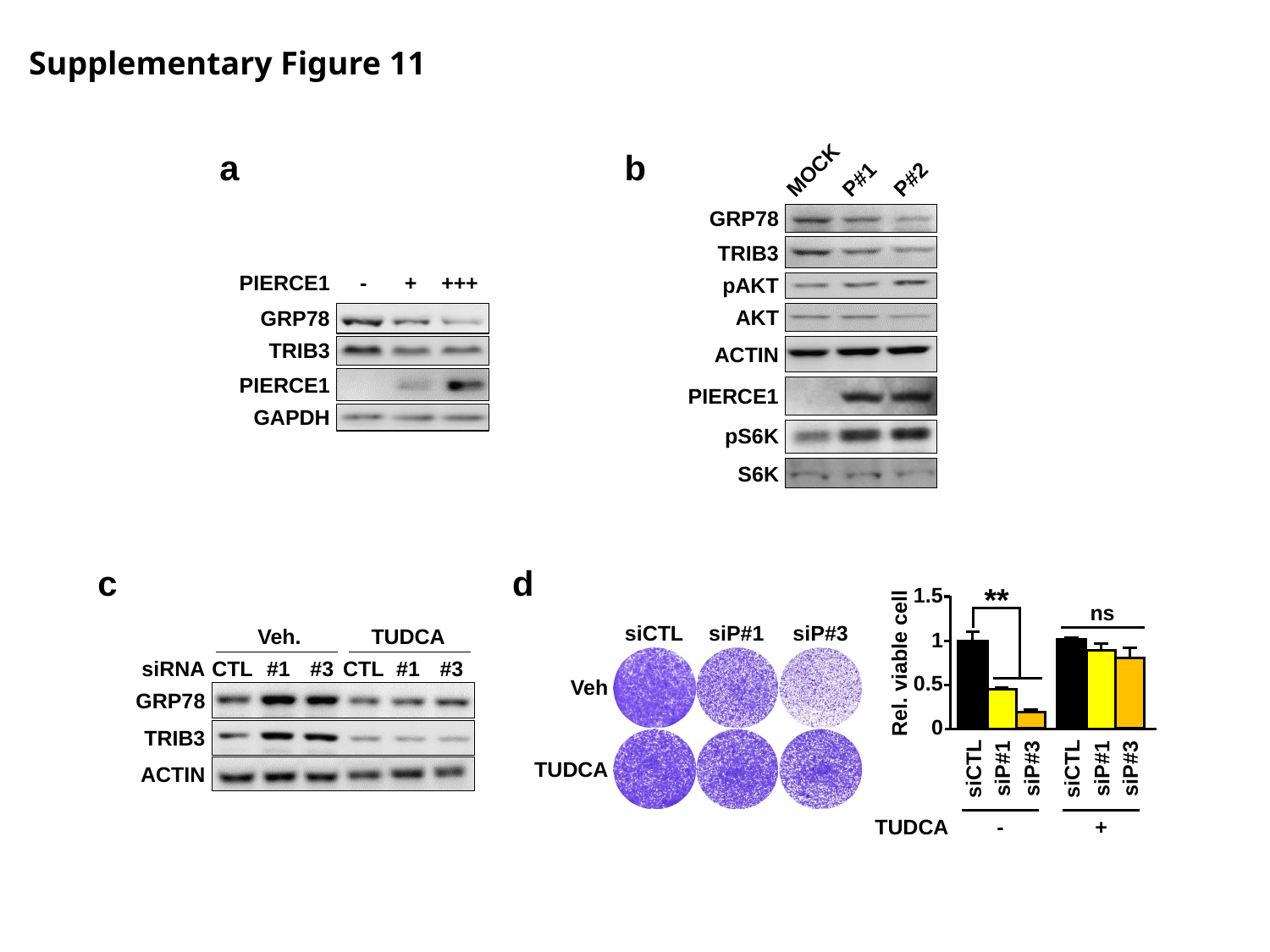

Supplementary Figure 11
a
b
MOCK
P#1
P#2
GRP78
TRIB3
pAKT
AKT
ACTIN
PIERCE1
pS6K
S6K
PIERCE1
-
+
+++
GRP78
TRIB3
PIERCE1
GAPDH
c
d
**
1.5
ns
1
Rel. viable cell
0.5
0
siCTL
siP#1
siP#3
siCTL
siP#1
siP#3
-
+
TUDCA
siCTL
siP#1
siP#3
Veh
TUDCA
Veh.
TUDCA
siRNA
CTL
#1
#3
CTL
#1
#3
GRP78
TRIB3
ACTIN

## Slide 12
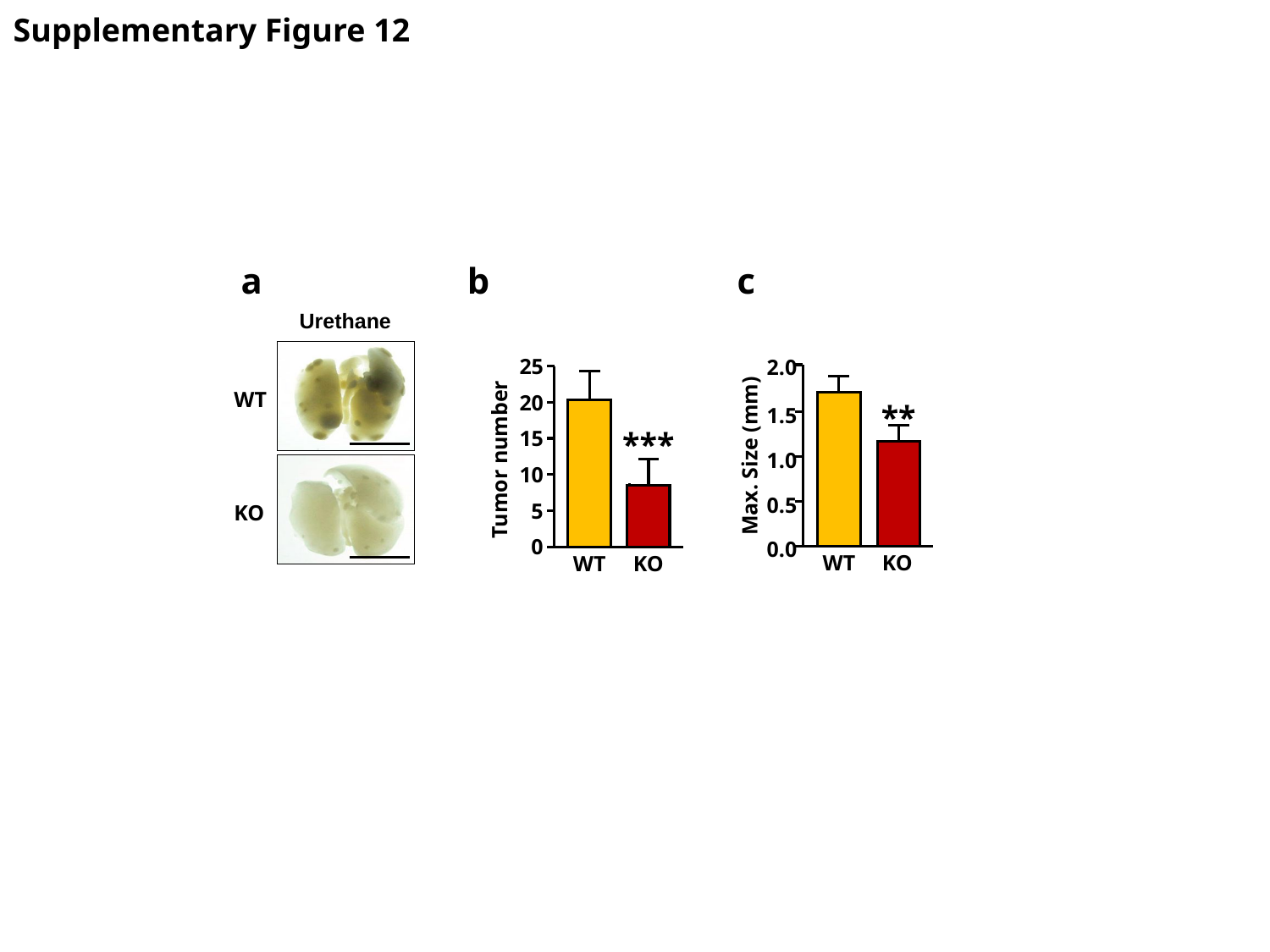

Supplementary Figure 12
a
b
c
Urethane
WT
KO
25
2.0
20
**
1.5
***
15
Max. Size (mm)
Tumor number
1.0
10
0.5
5
0
0.0
WT
KO
WT
KO

## Slide 13
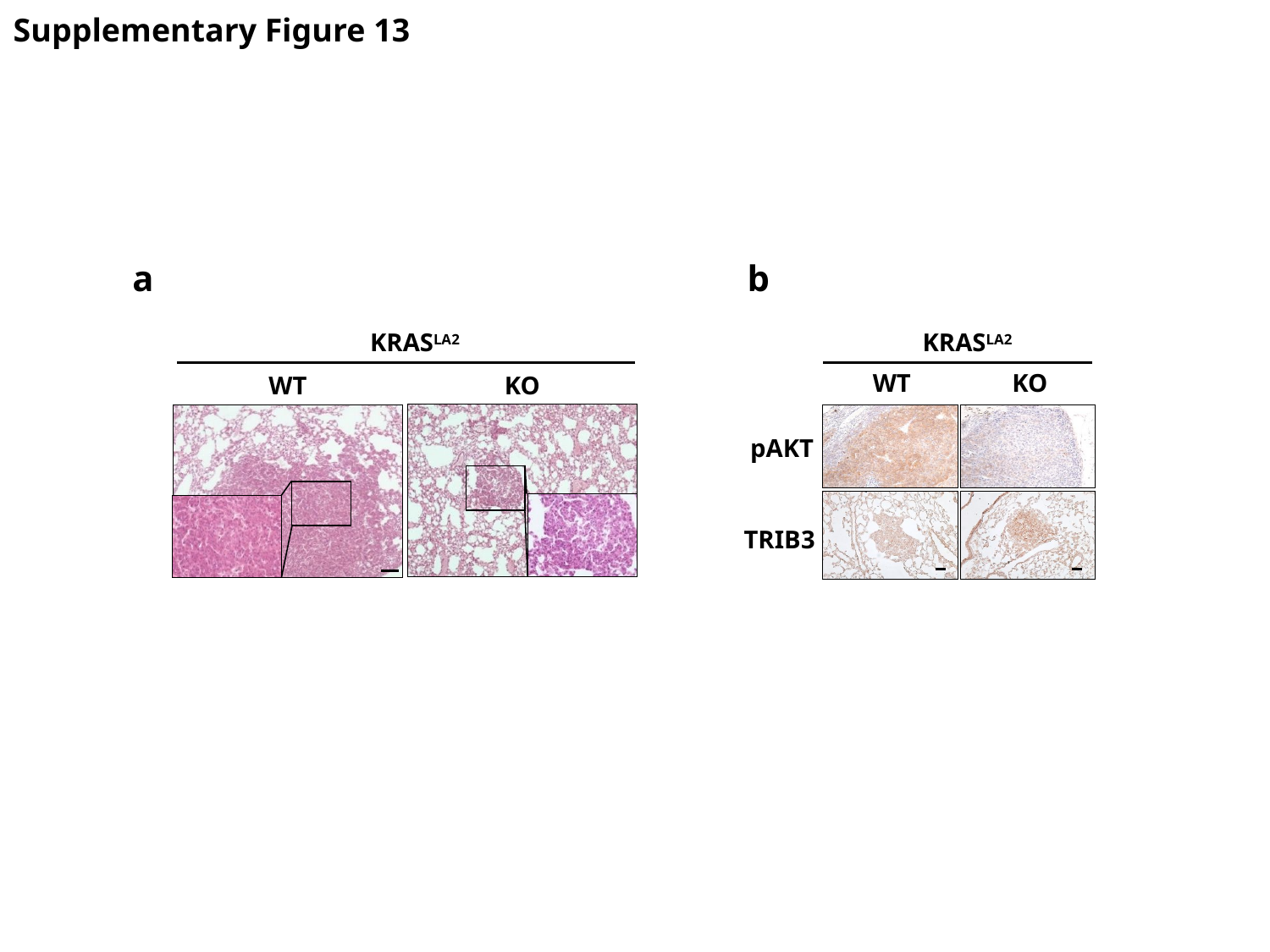

Supplementary Figure 13
a
b
KRASLA2
KRASLA2
WT
KO
WT
KO
pAKT
TRIB3

## Slide 14
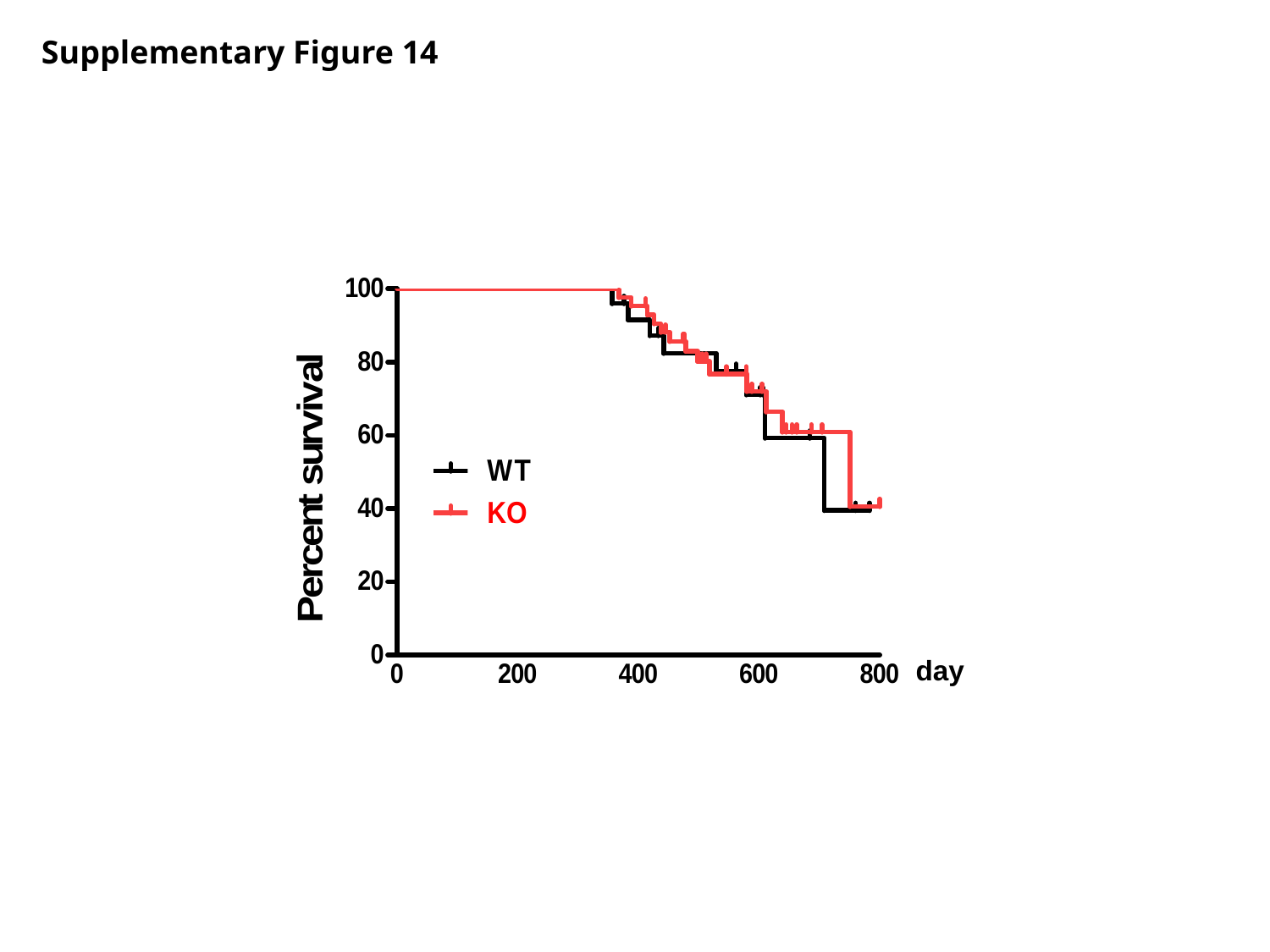

Supplementary Figure 14
day

## Slide 15
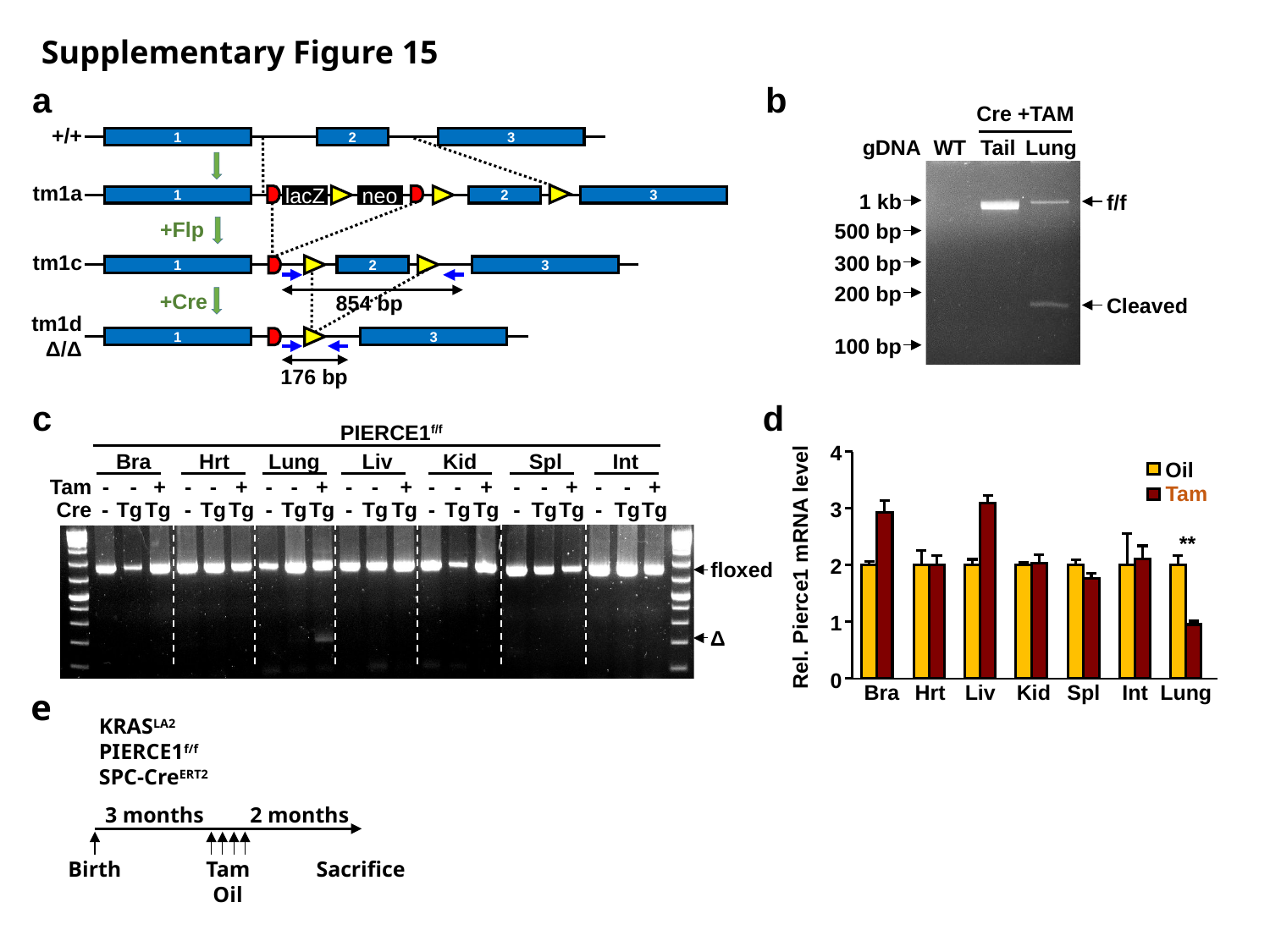

Supplementary Figure 15
a
b
Cre +TAM
+/+
1
2
3
gDNA
WT
Tail
Lung
tm1a
1
lacZ
neo
2
3
1 kb
f/f
+Flp
500 bp
tm1c
300 bp
1
2
3
200 bp
+Cre
854 bp
Cleaved
tm1d
Δ/Δ
1
3
100 bp
176 bp
c
d
PIERCE1f/f
4
Bra
Hrt
Lung
Liv
Kid
Spl
Int
Oil
Tam
-
-
+
-
-
+
-
-
+
-
-
+
-
-
+
-
-
+
-
-
+
Tam
Cre
-
Tg
Tg
-
Tg
Tg
-
Tg
Tg
-
Tg
Tg
-
Tg
Tg
-
Tg
Tg
-
Tg
Tg
3
**
Rel. Pierce1 mRNA level
floxed
2
1
Δ
0
Bra
Hrt
Liv
Kid
Spl
Int
Lung
e
KRASLA2
PIERCE1f/f
SPC-CreERT2
3 months
2 months
Birth
Tam
Oil
Sacrifice

## Slide 16
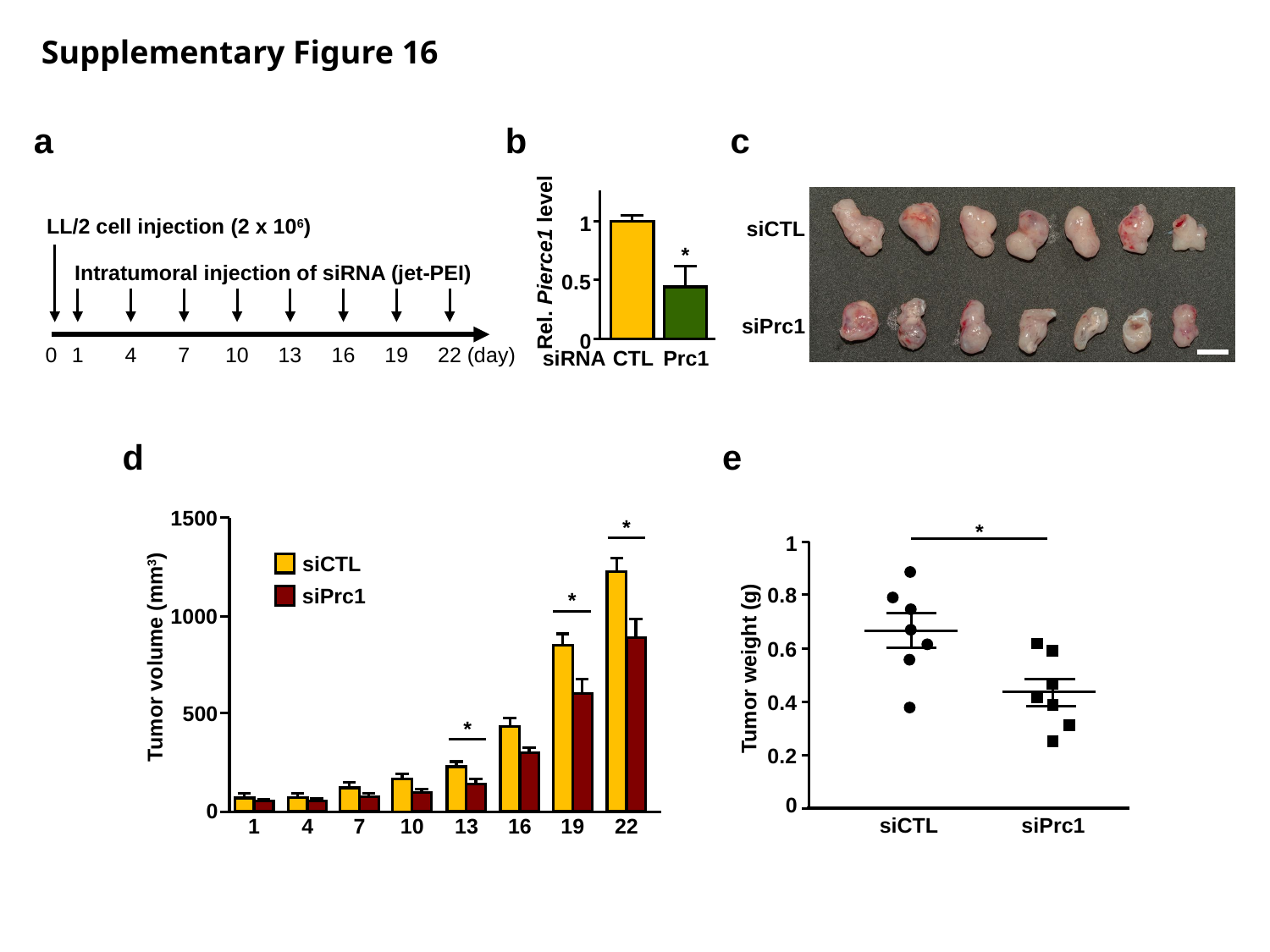

Supplementary Figure 16
a
b
c
LL/2 cell injection (2 x 10^6^)
siCTL
1
*
Rel. Pierce1 level
Intratumoral injection of siRNA (jet-PEI)
0.5
siPrc1
0
0
1
4
7
10
13
16
19
22
(day)
siRNA
CTL
Prc1
d
e
1500
*
*
1
siCTL
0.8
siPrc1
*
1000
0.6
Tumor volume (mm3)
Tumor weight (g)
0.4
500
*
0.2
0
0
siCTL
siPrc1
1
4
7
10
13
16
19
22

## Slide 17
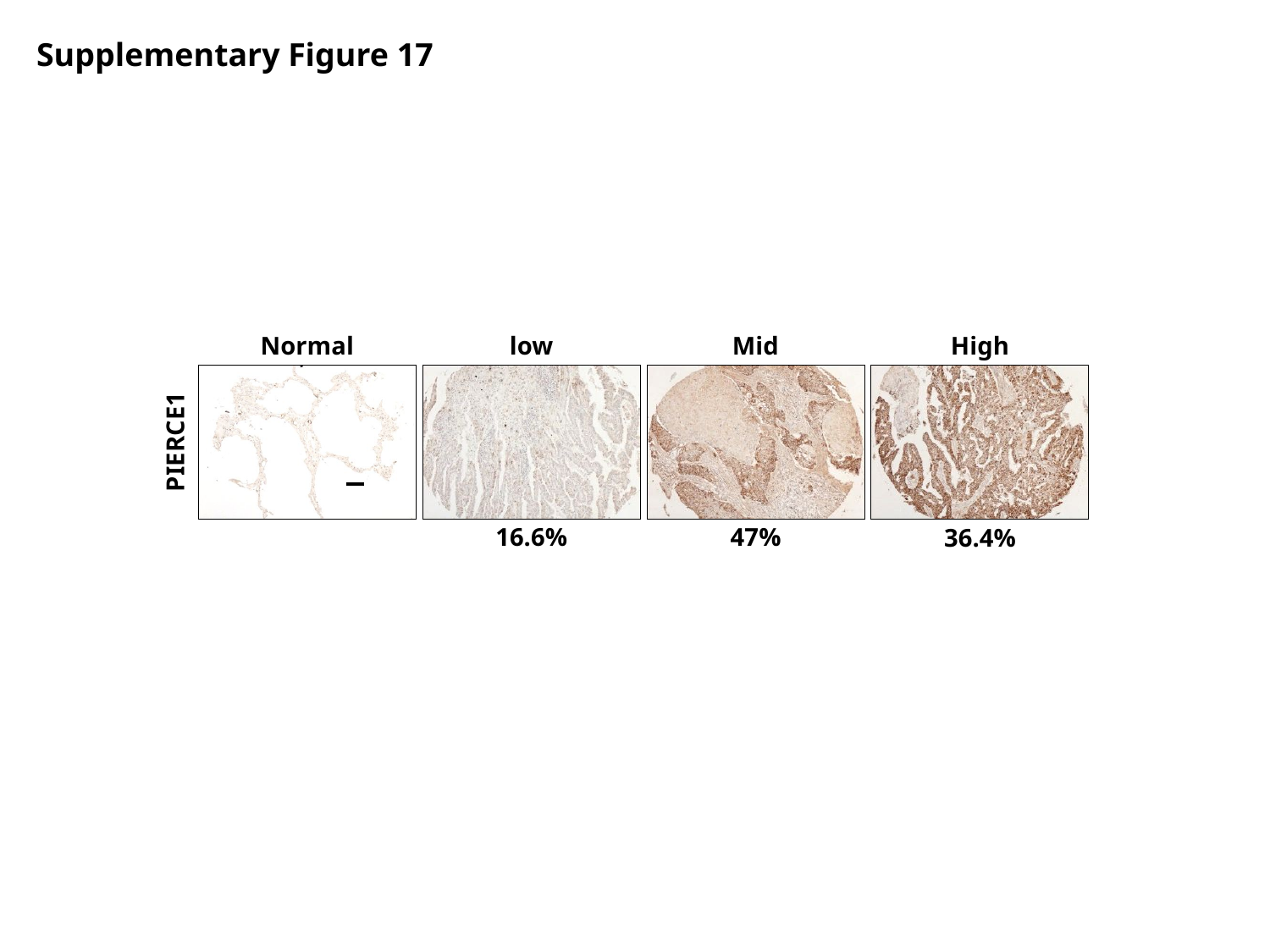

Supplementary Figure 17
Normal
low
Mid
High
PIERCE1
16.6%
47%
36.4%
